# Recent Advancements in the Diversification and Applications of Boron-Containing Compounds in Medicinal Chemistry †

**DOI:** 10.3390/ph18121798

**Published:** 2025-11-26

**Authors:** Marielle B. Frooman, Moinak K. Deb, Jaxon Peters, Sasha Leggett, Nitesh Sanghai, Nafees Zahra Rizvi, Devi Atukorallaya, Geoffrey K. Tranmer

**Affiliations:** 1 School of Chemistry and Biochemistry, Georgia Institute of Technology, Atlanta, GA 30332, USA; mariellef@gatech.edu; 2 College of Pharmacy, Rady Faculty of Health Sciences, University of Manitoba, Winnipeg, MB R3E 0T5, Canada; mdeb02@student.ubc.ca (M.K.D.); peter52@myumanitoba.ca (J.P.); leggett2@myumanitoba.ca (S.L.); 3 Department of Chemical and Biological Engineering, Faculty of Applied Science, University of British Columbia, Vancouver, BC V6T 1Z3, Canada; 4 Department of Oral Biology, Dr. Gerald Niznick College of Dentistry, Rady Faculty of Health Sciences, University of Manitoba, Winnipeg, MB R3E 0T5, Canada

**Keywords:** boron, boron-containing compounds, advanced drug delivery systems, drug design, biological molecules

## Abstract

Boron-containing compounds have made a significant impact on the field of medicinal chemistry since the discovery of Bortezomib (Velcade^®^), a dipeptide boronic acid approved by the FDA in 2003 for the treatment of multiple myeloma. Since then, over the last two decades, four more boron-containing drugs have been approved by the FDA: Tavaborole (Kerydin^®^), Ixazomib (Ninlaro^®^), Crisaborole (Eucrisa^®^), and Vaborbactam (in Vabomere^®^). These compounds are approved for treating conditions such as onychomycosis, multiple myeloma, and atopic dermatitis, as well as an Aβ-lactamase inhibitor approved in combination with meropenem for treating infections. Further, many organic molecules containing boron are in clinical trials. Additionally, boron-containing compounds play a crucial role in various biological processes. Boron’s Lewis acidity has been utilized for diverse applications, from targeting biological molecules to the synthesis of organic compounds and in advanced drug delivery systems. Recent progress in the advancement of boron-containing compounds has not stopped, and the further use of Boron is emerging day-by-day with the discovery of multifaceted applications. This review aims to highlight the recent advances made in the last decade in the drug design of boron-containing compounds and their therapeutic applications. Here, in this work, we have focused on the recent diversification and progress of boron-containing compounds in medicinal chemistry applications.

## 1. Introduction

Boron (B) is a group 13 metalloid element. It is distinct from other group 13 elements, having similar chemical properties to those of carbon (C) and silicon (Si). However, B is more electrophilic than both C and Si [1]. The ground state electronic configuration of B is 1s^2^2s^2^2p^1^. A B atom typically forms a trigonal geometry with sp^2^ hybridization and possesses the feature of electrophilicity because of the presence of a vacant P orbital. Due to six valence electrons, B is isoelectronic to carbocations. Additionally, the isoelectronic nature of C=C and B-N has spurred the use of B in drug design and medicinal chemistry [2,3]. The Lewis acid character of B has been immensely exploited in drug design and medicinal chemistry. Furthermore, B can also form a negatively charged tetravalent compound with sp3 hybridization, exhibiting a tetrahedral geometry. Additionally, the B center can be easily converted from a neutral trigonal planar sp^2^ to a tetrahedral sp^3^ hybridization under certain physiological conditions [4,5]. With a history spanning over 160 years, B has attracted the attention of medicinal chemists due to its wide range of applications, extending from drug design [6,7] to biochemistry [8,9] and further into advanced drug delivery systems [10,11]. Due to its stability and ease of synthesis, B has been utilized in various organic chemistry reactions for the formation of diverse chemical bonds, including hydroboration reactions (C-H and C-OH bonds), Suzuki–Miyaura coupling (C-C bonds), and the Chan–Lam coupling (C-N and C-O bonds). Among all Suzuki–Miyaura coupling reactions brought a revolution in the field of medicinal chemistry, due to several advantages which include the use of mild reaction conditions, easy commercial and cost-effective availability of boronic acid as reactants, and further its tolerance in wide reaction conditions, high conversion rates to products and mild conditions [12]. In the year 2014, after amide bond formation, the Suzuki coupling reaction was utilized as the most resourceful reaction by the pharmaceutical industries for the formation of carbon–carbon bonds with aromatic or heterocyclic groups [13].

Similarly to the utility in the organic synthesis, the biochemistry of B organic boron molecules arises from the ability of B to convert its trigonal planar geometry to tetrahedral geometry by accepting a lone pair of electrons from a biological nucleophile, generally an amino acid side chains in the protein biomolecule or from sugars of nucleic acids. Thus, organoborane compounds are one of the versatile organic molecules that are important in biological systems through their ability to form reversible covalent bonds with biomolecules having hydroxyl or amine groups. The result of the reversible tetrahedral complex is called borates, which is involved in covalent enzyme inhibition. This property of protein target engagement confers the chameleonic ability to the organoboron compound due to the change of uncharged (Sp^2^) hybridization to an anionic (Sp^3^) hybridization, essential for target engagement and inactivation of the enzyme [14]. Recently, the electrophilic B compounds have been designated as covalent warheads because of their ability to act as a reversible covalent ligation with the active site of nucleophiles in biomolecules for their inhibition [15]. Organoboranes like boronic acid also form reversible covalent complexes with sugars diols [16], amino alcohols [17], alkoxides [18], and hydroxamic acids [19].

The boronic acid-sugar (BA-S) covalent complex could be used as a biomarker in certain disease conditions like cancers, where carbohydrate-related antigens are found [20,21]. Numerous reports in recent times have exploited the use of boronic acids to recognize and sense carbohydrates using fluorescence as an endpoint for detection [22,23]. This is because of the use of boronic acid, which shows a change in fluorescence intensities upon binding with different sugar polyols [24,25,26]. Further, the boronic acids give an additional advantage of increased pharmacophore binding with amino acid residues, due to the presence of two hydroxyl groups, rendering four lone pair electrons and two hydrogen bond donors, thus offering six favorable opportunities for hydrogen bonding with different biomolecules. Furthermore, the enhanced binding with the target even in the presence of drug-resistant gene abnormalities [27,28]. In addition, boron has a unique property of emitting alpha particles under neutron spray bombardment, which forms a basis for using B in boron Neutron Capture Therapy (BNCT) for the treatment of cancers, overcoming the challenges of clinical oncology [29].

The potential of B as a therapeutic agent was realized with the discovery of bortezomib (Velcade^®^), in 2003, a first-in-class anticancer drug, approved by the Food and Drug Administration (FDA) as a proteasome inhibitor for the treatment of relapsed multiple myeloma and mantle cell lymphoma [30]. Since then, several other B-containing compounds have been approved by the FDA, which include tavaborole(onychomycosis) [31], Ixazomib (multiple myeloma) [32], crisaborole (atopic dermatitis) [33] and vaborbactam (bacterial infections) [34]. Further, several other B compounds are in clinical stages of development. It is noteworthy to mention the role of B as a heterogeneous green catalyst in driving various chemical reactions, thereby eliminating the hazards of using metal catalysts [35]. The last few decades have witnessed tremendous development in the potential of B in pharmaceuticals by exploring the chemical properties of B, hence making it a special element. Further research is warranted to uncover its future potential. In this comprehensive review, we have explored the latest advancements in the diversification of B in drug design and medicinal chemistry, as well as its role in advanced drug delivery systems.

## 2. Significance of This Work

This manuscript provides a brief overview of several recent advancements that have ushered in a new era and renewed hope in both drug discovery and drug delivery systems. First, researchers have engineered novel synthetic antibiotic analogues derived from a calcium-dependent antibiotic, activated by phenylboronic acid (PBA) instead of calcium ions. This research has, for the first time, demonstrated the role of boron in modulating the functions of drug molecules, opening new avenues for exploring boron’s role in drug discovery. Second, we presented a fully genetically encoded boronic-acid-containing designer enzyme with organocatalytic reactivity not achievable with natural or engineered biocatalysts. This research highlights how boronic acids could be used for genetic code expansion to create enantioselective enzymes. Third, we discussed recent research where boronic acid catalysts were used to form selective glycosidic bonds. This technique is valuable because it offers high selectivity and effectiveness for the “late-stage” modification of complex molecules without requiring extensive protecting groups. Fourth, we reviewed advances in the chemistry of Benzoxaborole, including the innovative concept of boron-containing compounds (benzoxaboroles) as prodrugs that undergo biochemical conversion to active drugs without relying on enzyme-dependent systems. This approach represents a new method in prodrug design and has been applied to developing drugs for conditions like tuberculosis. Additionally, the preclinical candidate DNDI-6148, a benzoxaborole for visceral leishmaniasis, a parasitic disease endemic in multiple regions worldwide, was discussed. Lastly, we briefly touched on several other groundbreaking advances in boron medicinal chemistry, including boron-containing compounds for treating fungal infections, Alzheimer’s disease, biological target engagement, novel drug chemotypes, potential hemiboronic acid naphthoids, non-β-lactam β-lactamase inhibitors against KPC-2 producers, arginase inhibitors, treatments for Chagas disease, liposome-encapsulated therapies for neutron capture therapy, strategies to combat resistant bacteria, and post-translational insertion of boron into proteins. Current progress with boron has shifted paradigms in drug discovery, illustrating the evolution of boron as both a promising medicinal agent and a drug delivery carrier. Furthermore, its role as a modulator of drug function and biological systems continues to advance.

**Significance statement: This manuscript emphasizes the transformative role of boron chemistry in advancing drug discovery and delivery, highlighting its potential as a versatile agent and a paradigm shift in developing innovative therapeutics and biological modulators.**

## 3. A Boron-Dependent Antibiotic

**Figure 1:**
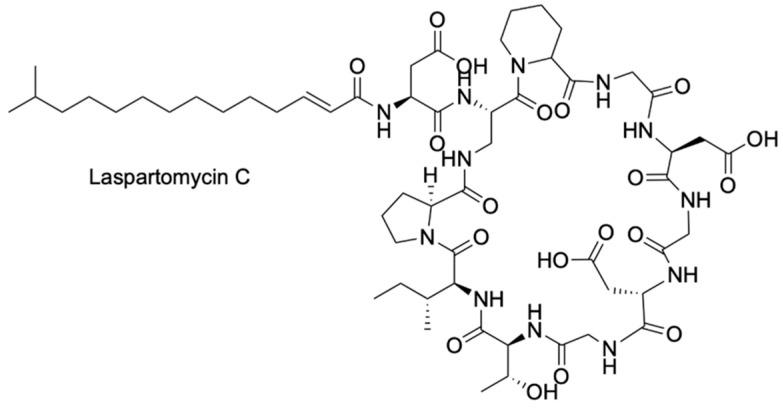
Structure of Laspartomycin C. Figures are created using ChemDraw (Version number 22.2.0).

**Figure 2:**
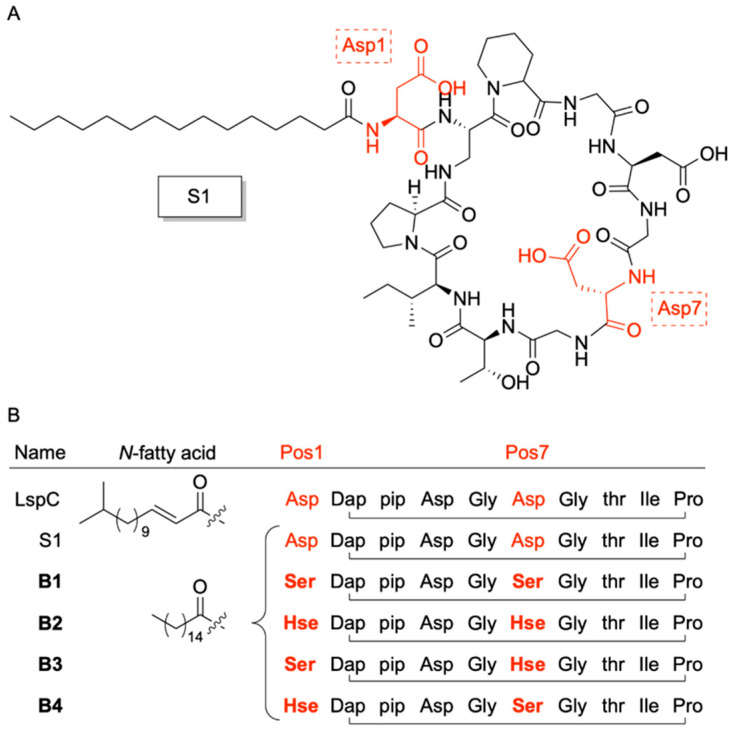
Synthetic LspC analogues: (**A**) Overall structure of LspC and its synthetic variants. S1 is identical to LspC with only minor differences in the fatty acyl chain. Four synthetic analogues (B1–B4) represent all possible combinations of Ser/Hse substitutions at residues 1 and 7 (marked in red). Structure of control S1 with Asp1 and Asp7 calcium-binding residues labeled in red (top). Serine and homoserine substitutions in analogues B1-B4 (bold) compared to those of the S1 control and LspC (bottom). (**B**) Amino acids are displayed in three-letter codes, wherein the capital and lowercase codes indicate the L-form or D-form, respectively. Figures are created using ChemDraw.

In 2023, Chiou et al. [36], reported the development of a novel class of boron-dependent antibiotics derived from Laspartomycin C (LspC), a cyclic lipopeptide recognized as a calcium-dependent antibiotic (CDA) (Figure 1). Initially isolated from *Streptomyces viridochromogenes*, LspC demonstrates notable antibacterial activity against a spectrum of Gram-positive pathogens, including methicillin-resistant *Staphylococcus aureus* (MRSA), vancomycin-intermediate *S. aureus* (VISA), vancomycin-resistant *S. aureus* (VRSA), and vancomycin-resistant *enterococci* (VRE) [37,38,39]. The advancement of boron-dependent analogues of LspC by the Chiou group presents a promising alternative to CDAs, which are currently limited in clinical efficacy due to the relatively low extracellular free calcium ion concentrations in humans (1.1–1.4 mM), a range that is insufficient to fully activate the majority of CDAs [40,41]. Interestingly, to overcome this limitation, Chiou and team substituted the aspartic acid (Asp) residues at positions 1 and 7 of LspC key residues involved in calcium coordination [42] with serine and homoserine, amino acids bearing hydroxyl side chains. All possible combinations of serine (Ser) and homoserine (Hse) were introduced at these positions, generating LspC analogues capable of binding phenylboronic acid (PBA) in place of calcium through esterification (Figure 2). This binding event at residues 1 and 7 induces conformational rearrangements that position the phosphate head group of the bacterial target molecule undecaprenyl phosphate (C_55_-P) in close proximity to the modified residues, thereby stabilizing the antibiotic’s active conformation [39].

Antimicrobial assays using *Bacillus subtilis* were conducted to evaluate the bactericidal activity of four synthetic LspC analogues (B1–B4) (Table 1). Three of the analogues (B1, B3, and B4) exhibited potent antimicrobial effects, with minimum inhibitory concentrations (MICs) comparable to that of the control compound S1, a structurally similar LspC variant with no Asp substitutions. Among the active analogues, B1 demonstrated the most promising profile, exhibiting the lowest MIC, while requiring only a single equivalent of PBA for activation. Notably, while Kleijn et al. [39]. reported that native LspC required a 5 mM calcium ion concentration to achieve a MIC of 4 µg mL^−1^, synthetic analogue B1 achieved the same MIC with just 0.82 mM of supplemented PBA, indicating a significant improvement in activation efficiency.

Antibiotic activity studies conducted by Chiou revealed two key findings: (i) in the presence of PBA, the MICs of the three active synthetic analogues (B1, B3, and B4) were unaffected by calcium ion concentration, and (ii) in the absence of PBA, calcium-dependent bacterial inhibition by these analogues was negligible. Collectively, these results highlight the high selectivity and PBA-dependent activation of the synthetic analogues, underscoring their robustness and potential as tunable antimicrobial agents.

The antimicrobial activity of B1 was further evaluated against a panel of fourteen Gram-positive bacterial species, with exceptionally robust bactericidal capacity demonstrated against methicillin-resistant and vancomycin-resistant strains. These findings position B1 as a promising candidate to fulfill the roles of anti-MRSE (*methicillin-resistant Staphylococcus epidermidis*) and anti-VRE (*vancomycin-resistant Enterococcus*) calcium-dependent antibiotics (CDAs), such as daptomycin and malacidin A [41,43]. Additionally, the cytotoxic concentrations of B1 and PBA were found to be nearly two orders of magnitude higher than their respective MIC range across the tested Gram-positive species, supporting the potential of B1 as a safe and effective candidate for clinical development.

**Table 1:** Comparison of minimum inhibitory concentrations (MICs) of LspC analogues with common CDAs [36,39,44]. MICs of CDAs Laspartomycin C, Daptomycin, Friulimicin B, and LspC analogue S1 with *S. aureus* in the presence of 5 mM Ca^2+^ are listed. Calcium-independent LspC analogues B1, B2, B3, and B4 are listed with their MICs of *B. subtillis* when supplemented with 0.82 mM phenyl boronic acid (PBA). “>” denotes no noticeable growth suppression at the highest concentration tested (128 μg/mL (=103, 101, 106, 104, 105, and 105 μM for LspC)).

Name	Pathogen	Supplement	MIC µg/mL	Reference
Laspartomycin C	*Staphylococcus aureus*	5 mM Ca^2+^	4	[39,44]
S1	*Bacillus subtillis*	5 mM Ca^2+^	4	[36]
B1	*Bacillus subtillis*	0.82 mM PBA	3.3	[36]
B2	*Bacillus subtillis*	0.82 mM PBA	>	[36]
B3	*Bacillus subtillis*	0.82 mM PBA	6.5	[36]
B4	*Bacillus subtillis*	0.82 mM PBA	6.5	[36]
Daptomycin	*Staphylococcus aureus*	5 mM Ca^2+^	0.25	[39,44]
Friulimicin B	*Staphylococcus aureus*	5 mM Ca^2+^	2	[44]

**Figure 3:**
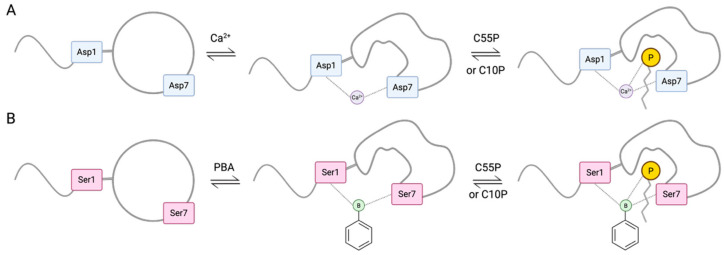
Schematic illustration of calcium or phenyl boronic acid (PBA)-induced conformational changes of LspC and B1. (**A**) Coordination between the Asp1 and Asp7 residues of LspC with Ca^2+^ induces a conformational change that allows binding of the target phosphate ligand. (**B**) The modified Ser1 and Ser7 residues of B1 undergo a comparable conformation change when bound to PBA, thereby permitting complex formation with the target phosphate head (**B**). Created in BioRender. Frooman, M. (2025) https://BioRender.com/i62et37.

While these findings underscore the potential of B1 in diverse antimicrobial applications, they also offer valuable insights into its mechanism of action (MOA). Specifically, the data suggests that B1’s antimicrobial activity is selective for Gram-positive bacteria, with no observable effect against Gram-negative species. In their initial studies, Kleijn et al. proposed that LspC disrupts cell wall biosynthesis through the sequestration of bacterial C_55_-P [39]. To investigate whether the PBA-dependent LspC analogues operate via a similar MOA, Chiou and colleagues first performed molecular dynamics (MD) simulations of each analogue in complex with PBA and a stabilized C_55_-P surrogate, C_10_-P. These simulations demonstrated that the active conformations of the analogues were stable under physiological conditions. Combined with inhibition and thin-layer chromatography (TLC) assays, the computational results support the hypothesis that the analogues may share a conserved MOA with native LspC. Further characterization using high-resolution native electrospray ionization mass spectrometry (HR-NESI-MS), as well as phosphorous (31P) and boron (11B) NMR, confirmed that B1 forms a ternary complex with the phosphate head group of the target molecule in the presence of PBA (Figure 3). Notably, this complex formation was largely unaffected by calcium, reinforcing the specificity and robustness of the PBA-dependent mechanism.

Further, Chiou successfully synthesized and evaluated four boron-dependent antibiotics that demonstrate strong potential as alternatives to calcium-dependent agents. Their antimicrobial efficacy was shown to depend on the specific placement of homoserine (Hse) and serine (Ser) substitutions at positions 1 or 7 of the cyclic peptide backbone derived from the LspC parent compound. It should be noted that while the synthetic analogues’ adoption of the active structure was dependent on the concentration of PBA, native LspC binds calcium in a 1:2 stoichiometric ratio, suggesting that the boron-dependent analogues may still interact with calcium in a non-activating manner. However, calcium was found to have little to no effect on the MICs of the analogues, which are comparable to that of LspC, highlighting their functional independence from calcium for activation. Considering the advantages of boron in medicinal chemistry, the synthesized boron-dependent analogues by Chiou present a promising alternative to current CDA systems. However, with the ongoing discovery and synthesis of more effective CDA alternatives, it is essential to advance the boron-dependent analogues in order to remain viable substitutes for existing therapies.

## 4. Boron Catalysis in a Designer Enzyme

Engineered enzymatic biocatalysis has garnered considerable attention in synthetic chemistry for its potential to transcend the limitations of natural enzymes, particularly in enabling non-specific enantioselective transformations across structurally diverse substrates. Boronic acids (BAs) have emerged as promising catalytic motifs due to their ability to participate in multiple activation pathways and interact with a broad range of functional groups [45]. However, current enantioselective BA-based catalysts generally rely on auxiliary chiral scaffolds, such as thiourea or amine derivatives, to impart stereocontrol [46,47]. Additionally, the incorporation of aryl substituents is often necessary to stabilize the boron center, which restricts access to more elaborate designs involving vicinal chiral centers and limits the broader application of boron-mediated asymmetric catalysis [45].

**Figure 4:**
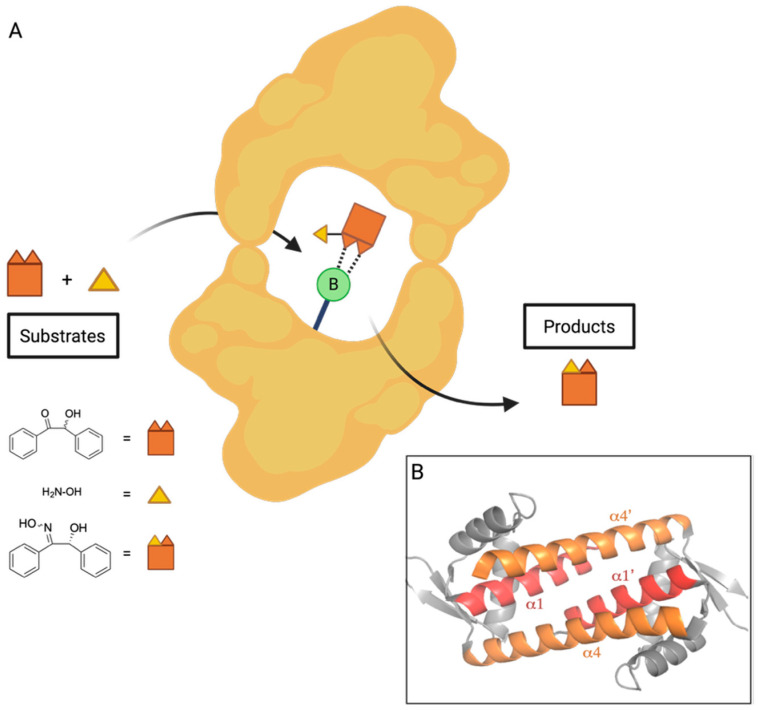
(**A**) Schematic representation of catalytic activity within the designer LmrR enzyme. The LmrR variant containing a M89pBoF mutation catalyzes the reaction of racemic benzoin and hydroxylamine in an enantioselective fashion. (**B**) Front view of the dimeric LmrR protein variant structure containing a M89pBoF mutation (PDB ID: 8QDF) [48]. The binding interface consisting of α-helices 1 and 4 of each monomer is shown. Helices belonging to each monomer in the pair are labeled as α1 and α4 or α1′ and α4′, respectively. Created in BioRender. Frooman, M. (2025) https://BioRender.com/sl30f8z.

In a recent advance toward the development of an enantioselective BA-based catalyst, Longwitz et al. reported a viable biosynthetic strategy based upon protein engineering [48]. Using stop codon suppression, they successfully incorporated para-boronophenylalanine (pBoF), a noncanonical amino acid bearing a boronic acid moiety, into key binding residues of the Lactococcal multidrug resistance regulatory protein (LmrR). LmrR is a structurally adaptable, dimeric transcriptional regulator known for its hydrophobic ligand-binding interface, formed primarily by α1 and α4 helices [49] (Figure 4). By substituting six residues along these helices with pBoF, the authors generated a library of LmrR variants featuring boronic acid functionalities within the protein’s active site.

**Figure 5:**
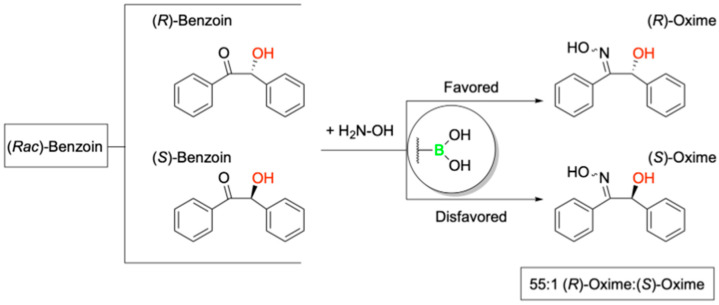
The designer boronic-acid (BA)-dependent oxime synthase (BOS) enzyme catalyzes the reaction of racemic benzoin and hydroxylamine, selecting for the (R)-oxime product in a 55:1 ratio to its (S)- enantiomer. For simplicity, only the BA moiety of the pBoF residue in the designer enzyme is shown. Figures are created using ChemDraw.

Further, to evaluate the stereoselective catalytic potential of these engineered LmrR variants, the authors employed a racemic mixture of benzoin in the presence of hydroxylamine, demonstrating preferential conversion of the (*R*)-enantiomer to its corresponding oxime (Figure 5).

**Figure 6:**
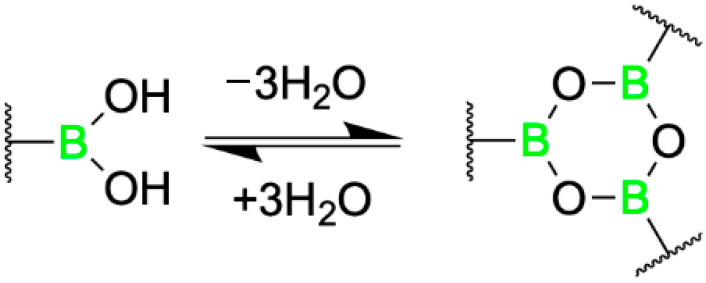
Conversion of boronic acid (BA) into the respective six-membered boroxine ring. Through an equilibrium of dehydration and hydration, three BA combine to form the resulting boroxine. Figures are created using ChemDraw.

This transformation, facilitated by BA-mediated dehydration, was proposed to proceed via a pseudo-five-or-six-membered ring intermediate under basic conditions, as Simionatto et al. established for boronic acid-catalyzed reactions involving α-hydroxy aldehydes [50]. Longwitz and team employed a combination of high-resolution mass spectroscopy (HRMS), ^11^B NMR, and X-ray crystallography to verify their hypothesis. Ultimately, all three techniques validated that, in the absence of substrate, the boron center of BOS predominantly exists in its low-valent boronic acid form, with partial conversion into its corresponding six-membered boroxine ring via dehydration (Figure 6).

**Figure 7:**
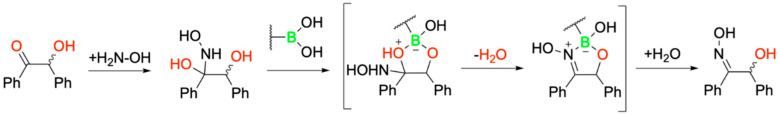
Scheme of BOS-mediated catalysis. Hydroxylamine performs a nucleophilic attack on the electrophilic carbonyl moiety of benzoin. In the presence of the pBoF residue of the mutant LmrR enzyme, a tetrahedral boronate ester intermediate is formed. Through a dehydration reaction, this intermediate undergoes a rearrangement, which, upon subsequent rehydration, yields the (R)-oxime product. Figures are created using ChemDraw.

Following nucleophilic attack of representative a-hydroxy ketones by hydroxylamine, the resulting transient vicinal diol was shown to coordinate with the boronic acid center of BOS, forming a tetrahedral, sp^3^ hybridized cyclic boronate ester intermediate, as evidenced by HRMS and ^11^B NMR (Figure 7). The observed catalytic activity is attributed to electrophilic activation of the a-hydroxy ketone by the BA moiety, while the observed stereoselectivity highlights the potential of pBoF-containing proteins as scaffolds for asymmetric catalysis, expanding the utility of boronic acids in biocatalytic applications.

Among all variants tested, the incorporation of pBoF at position 89 of the LmrR protein scaffold yielded the most effective catalyst for enantioselective transformation. This designer enzyme, termed the boronic-acid-dependent oxime synthase (BOS), efficiently catalyzed the reaction of benzoin with hydroxylamine to produce the resulting (*R*)-oxime with high stereoselectivity, achieving a diastereomeric ratio of 13:1 in favor of the (*R*)-enantiomer. Notably, control experiments using LmrR variants containing structurally related, non-boronic amino acids at the same position, as well as reactions using free pBof, exhibited neither catalytic activity nor enantioselectivity. These results suggest a synergistic interaction between pBoF and the LmrR scaffold that is essential for the observed stereoselective catalysis by BOS.

Building on the observation that additional residues within the BOS binding groove contributed synergistically with the pBoF residue to enzymatic activity, Longwitz aimed to further enhance enantioselective catalysis through binding site optimization. This was achieved via multiple rounds of directed evolution using site-saturation mutagenesis (SSM). The resulting optimized variant, bearing the mutations F93E, M8H, and A92L in combination with M89pBoF, exhibited significantly improved stereoselectivity, producing the (R)-oxime with an enantiomeric ratio of up to 146:1. This enhanced catalyst, designated BOS_EHL (M89pBoF_F93E_M8H_A92L), maintained its high enantioselectivity across a broad range of substituted benzoin derivatives. Further, repeating the HRMS, ^11^B NMR, and X-ray crystallography studies with BOS_EHL yielded analogous results to those of BOS, suggesting a conserved catalytic mechanism between the parent and evolved enzymes. Together, these data validate the authors’ initial mechanistic hypothesis based on preliminary related works by Simionatto [50].

**Figure 8:**
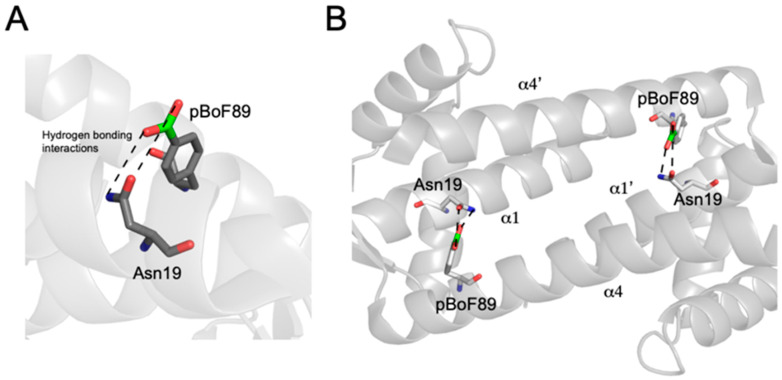
Hydrogen bonding interactions between Asn19 of the α1 helix and pBoF89 of the α4 helix maintain the structural integrity of the BOS binding groove. This stabilizing force is mirrored between the dimers. (**A**) Close-up of hydrogen bonding interaction. (**B**) Front view of interactions spanning the hydrophobic BOS binding groove. Figures are created using Pymol (v2.5.7).

To further explore the impact of the BOS protein environment on the BA’s ability to perform stereoselective catalysis, Longwitz analyzed the crystal structure of BOS, which revealed hydrogen-bonding interactions between the central boronic acid moiety and the nearby Asparagine 19 residue (Figure 8). This intramolecular interaction proved critical to maintaining the shape of the designer enzyme’s binding groove. Further, both the nitrogen and oxygen of the amide side chain of N19 interact with the boronate ester intermediate formed during the reaction of benzoin and hydroxylamine, likely providing stability to this key intermediate. The F93E, M8H, and A92L substitutions of BOS_EHL similarly improve the tightening interactions of the α1 and α4 helices of the binding groove. Interestingly, the authors note the negative influence of the phenylalanine residue at position 93 on the catalytic activity of BOS, but as it does not interact directly with the boron center, its role was not further explored [48].

Taken together, the findings of Longwitz et al. [48]. highlight the strong potential of boron-mediated catalysis for expanding the landscape of enantioselective transformations. The successful engineering of a boron-containing enzyme underscores the feasibility of developing new-to-nature catalysts that address limitations in nature’s existing catalytic toolkit. Notably, the study also acknowledges a key limitation: even the optimized BOS_EHL catalyst failed to convert certain substrates enantioselectively, particularly those incapable of forming the critical transient vicinal diol intermediate. This insight not only clarifies the current boundaries of the system but also provides a valuable roadmap for future work in xenobiotic catalysis.

## 5. Boron-Mediated Aglycon Delivery

Glycosidic linkages play a key role in the formation of polysaccharides and glycoconjugates, including glycoproteins and glycolipids. Naturally occurring glycosides are of particular interest in medicinal chemistry due to their diverse bioactive properties, including anticancer [51,52], antimicrobial [53,54], antifungal [55], antidiabetic [56,57], and cardioprotective [58,59] effects. Of the various types of glycosidic bonds, *O*-glycosidic linkages are among the most prevalent in nature.

**Figure 9:**
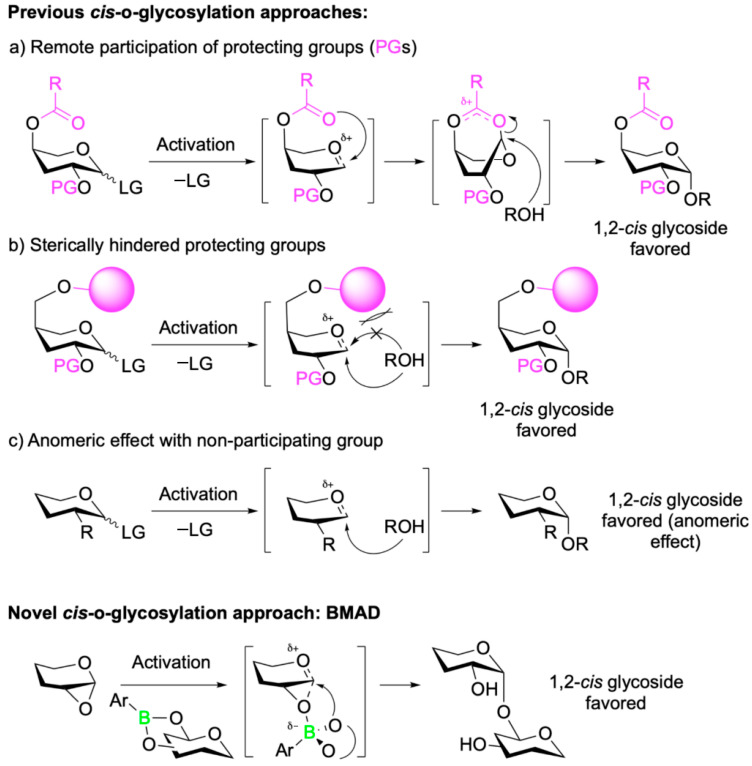
Strategies historically employed to achieve 1,2-cis glycosylations and novel boron-mediated aglycon delivery (BMAD) approach. (**a**) Remote participation of protecting groups, both structurally and electronically, favors the formation of 1,2-*cis*-glycosides. (**b**) Steric hindrance from protecting groups favors a pseudo-axial attack, which is more likely to occur in 1,2-*cis*-glycosides. (**c**) Anomeric effects from heteroatoms of non-participating groups favor 1,2-*cis*-glycosides. Protecting groups (PGs) are shown in pink. The novel cis-glycosylation BMAD approach utilizes an aryl boronic ester to form a tetrahedral intermediate, which ultimately yields the 1,2-cis-glycoside with high regio- and stereoselectivity. Figures are created using ChemDraw.

For synthetic chemists, the formation of 1,2-*trans*-*O*-glycosidic linkages is well established, with numerous approaches that offer high yields and excellent stereocontrol [60,61]. In contrast, the stereoselective synthesis of 1,2-*cis*-*O*-glycosidic linkages has historically posed greater challenges. However, significant progress over the past two decades has led to the development of several strategies that enable stereoselective access to these *cis*-linkages [62,63,64,65]. Despite these advances, many of the existing methods rely heavily on extensive protection and deprotection steps (Figure 9). These multistep protocols often increase production time, cost, and labor requirements, while reducing atom economy and falling short of green chemistry standards.

**Figure 10:**
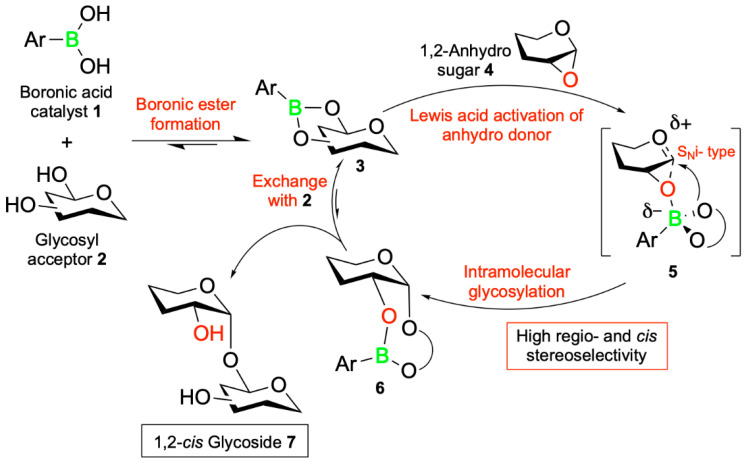
Boron-mediated aglycon delivery (BMAD) catalytic cycle. Through an esterification reaction between a boronic acid catalyst and glycosyl acceptor, a trigonal planar boronic ester is formed that can undergo nucleophilic attack by a 1,2-anhydro sugar. The resulting tetrahedral intermediate proceeds to perform an intramolecular nucleophilic substitution and intramolecular glycosylation that yields a 1,2-*cis*-glycoside. The cycle can continue with excess glycosyl, which binds to the recycled aryl boronic acid catalyst. Figures are created using ChemDraw.

In their recent study, Isozaki et al. [66] address these limitations by introducing a novel catalytic glycosylation method that bypasses the need for extensive protecting group chemistry. Their strategy, termed boron-mediated aglycon delivery (BMAD), enables the regio- and stereoselective formation of 1,2-*cis*-*O*-glycosidic linkages using unprotected sugars bearing free hydroxyl groups (Figure 9). This method leverages the reversible esterification reaction between the boronic acid catalyst **1** and the *cis*-1,2 or 1,3 diol moiety on the glycosyl acceptor **2** to form the boronic ester intermediate **3.** This intermediate activates the 1,2-anhydrosugar donor **4** for a late-stage S_N_i-type intramolecular glycosylation via the boronate ester transition state **5**. The resulting boronic ester **6** undergoes an exchange with the cis-diol **2** to complete the catalytic cycle, yielding the 1,2-*cis*-glycoside product **7** with high regio- and stereoselectivity (Figure 10).

**Figure 11:**
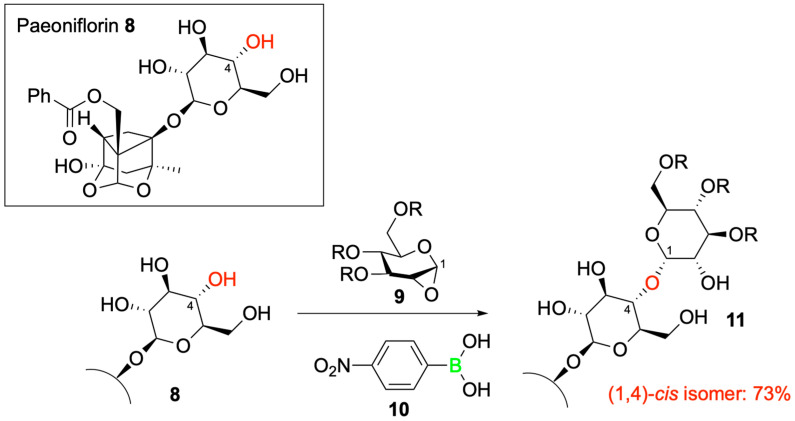
Late-stage functionalization of Paeoniflorin using the BMAD approach. In the presence of the aryl boronic acid catalyst **10**, the reaction of aglycon **9** with Paeoniflorin produces a 73% yield of the 1,4-cis isomer of the glycosylated Paeoniflorin. Figures are created using ChemDraw.

To demonstrate the utility of their BMAD strategy, Isozaki and his grouppplied this novel catalytic approach to the late-stage functionalization of the model glycosyl acceptor paeoniflorin **8,** a structurally complex molecule bearing multiple free hydroxyl groups. Remarkably, the authors were able to predict and achieve regioselective glycosylation at the C4 hydroxyl position, yielding the desired 1,2-*cis*-glycoside in high stereoselective yield using anhydrosugar **9** and BA **10** (Figure 11). This application underscores the potential of BMAD to selectively functionalize complex, unprotected substrates, thereby eliminating the need for extensive protection and deprotection protocols and thus enhancing synthetic efficiency and sustainability.

**Figure 12:**
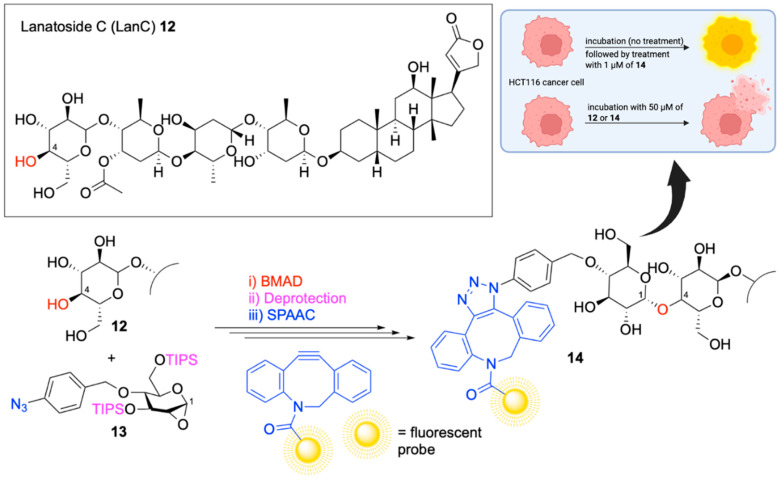
Scheme depicting a translational application of boron-mediated aglycon delivery (BMAD) with the anticancer compound Lanatoside C (LanC). Through a BMAD reaction between LanC with the azide-functionalized anhydrosugar **13** and subsequent deprotection, LanC can undergo a strain-promoted azide–alkyne cycloaddition with a dibenzocyclooctyne fluorescent probe. The resulting fluorescently labeled LanC retains its anticancer properties, as demonstrated by MTT assays with HCT116 human colon cancer cells. Figures are created using BioRender and ChemDraw. Created in BioRender. Frooman, M. (2025) https://BioRender.com/bsbu7u6.

Having validated their novel approach to *cis*-glycosylation, Isozaki and colleagues [66], explored its practical application in chemical biology. While numerous methods exist for protein labeling, glycosides often pose a greater challenge due to their high density of hydroxyl groups, which can complicate regioselectivity and compromise molecular functionality. Nevertheless, the ability to selectively label glycosides remains essential for probing complex carbohydrate-containing biological systems. To demonstrate the utility of the BMAD strategy in this context, the authors targeted the glycoside Lanatoside C (LanC), a compound with established inotropic [67] and anticancer [68,69] activity, for late-stage functionalization and fluorescent labelling. Applying BMAD with LanC **12** and 1,2-anhydrosugar **13**, they successfully introduced a cis-glycosidic linkage, which they followed with deprotection and a strain-promoted azide–alkyne cycloaddition (SPAAC) between the p-azidobenzyl group of **13** and a dibenzocyclooctyne (DBCO)-functionalized fluorescent probe. The desired fluorescently labeled (1,4)*-O*-glycoside product **14** was obtained in good 1,2-*cis* stereoselective yield. Importantly, MTT assays conducted on HCT116 human colon cancer cells revealed that the fluorescently labeled LanC retained its anticancer activity (Figure 12). Furthermore, using confocal fluorescence microscopy and affinity pulldown assays in the presence and absence of the labeled LanC, the authors were able to identify its protein target. These findings not only confirm the compatibility of BMAD with biologically active glycosides but also highlight its broader potential as a tool for chemical biology and target identification.

**Figure 13:**
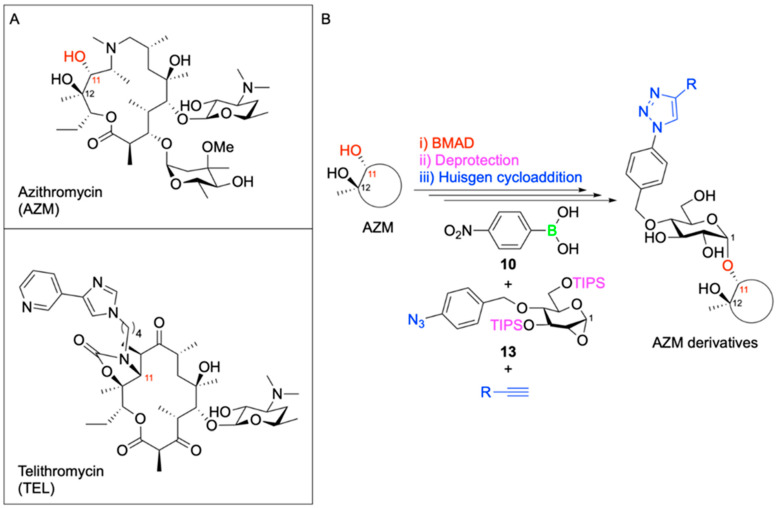
Due to increasing antibiotic resistance in pathogens, macrolide antibiotic azithromycin (AZM) is a candidate for chemical modifications inspired by previous studies with the macrolide antibiotic telithromycin (TEL). (**A**) Structures of AZM and TEL. Both macrolides contain at least one deoxy sugar moiety. (**B**) Scheme depicting the synthesis of AZM derivatives via late-stage BMAD reactions. Following the BMAD reaction between AZM and the azide-functionalized anhydro sugar **13**, a Huisgen cycloaddition enabled the synthesis of a library of various glycosylated AZM derivatives. Figures are created using ChemDraw.

A second application of the BMAD strategy involved the selective chemical modification of existing antibiotics to address emerging resistance mechanisms. The macrolide antibiotic azithromycin (AZM, Figure 5) remains a first-line therapy for pulmonary infections caused by nontuberculous mycobacteria (NTM). However, the growing prevalence of antibiotic-resistant NTM strains, such as macrolide-resistant *Mycobacterium avium* and *Mycobacterium intracellulare*, necessitates the development of alternative treatments. Drawing from previous structural studies of the related macrolide antibiotic telithromycin (TEL, Figure 13) and its interactions with NTM and other bacterial ribosomes [70]. Further, Isozaki and colleagues hypothesized that introducing a novel side chain at the C11 position of AZM could enhance ribosomal binding and potentially circumvent resistance mechanisms.

To test their hypothesis, the authors employed their BMAD approach to attach various substituted sugar moieties to the 1,2-diol motif comprising C11 and C12 of the AZM macrolide ring. Following deprotection and subsequent Huisgen 3 + 2 cycloaddition, a diverse library of glycosylated AZM analogues was synthesized (Figure 13). The BMAD reaction exhibited excellent regioselectivity and consistent 1,2-*cis*-stereoselectivity across a range of sugar donors.

In total, 13 novel AZM analogues were synthesized. These compounds were evaluated through minimum inhibitory concentration (MIC) assays against both wild-type and macrolide-resistant *M. avium* and *M. intracellulare* strains, in parallel with AZM and TEL. While AZM displayed potent activity against wild-type strains (MIC < 1 μg/mL), it was ineffective against macrolide-resistant isolates (MIC > 32 μg/mL). TEL showed comparable activity against wild-type NTM (MIC ≤ 1 μg/mL), moderate activity against resistant *M. intracellulare* (MIC = 32 μg/mL), and strong activity against resistant *M. avium* (MIC = 2 μg/mL).

Among the synthesized analogues, compound **KU13**, featuring a pyridine substituent at C11, demonstrated potent bactericidal activity across both wild-type and resistant NTM strains. **KU13** inhibited *M. avium* at 0.25 μg/mL and *M. intracellulare* at 0.125 μg/mL in wild-type strains, while maintaining activity against macrolide-resistant *M. avium* (MIC = 1 μg/mL) and *M. intracellulare* (MIC = 8 μg/mL). Additionally, in an evaluation of translational inhibitory activity of AZM and KU13 using an in vitro reconstituted, cell-free translation system, KU13 achieved complete translational inhibition at a dose of only 200 nM, half of the dose required for complete inhibition by AZM. These results highlight the potential of BMAD-enabled diversification to improve antibiotic efficacy against drug-resistant pathogens through ribosomal interference.

**Figure 14:**
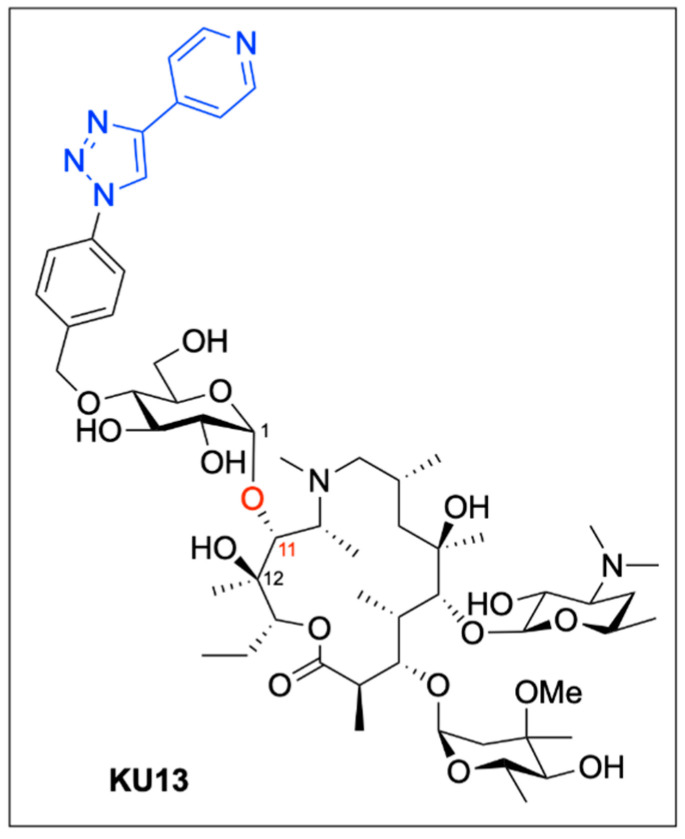
Structure of KU13, a novel macrolide antibiotic. Figures are created using ChemDraw.

To gain mechanistic insight into this improved activity, the authors employed single-particle cryogenic electron microscopy (cryo-EM) to visualize the structure of the **KU13**-bound *Mycobacterium tuberculosis* 70S ribosome (Figure 14). The cryo-EM structure revealed that **KU13** occupies the nascent peptide exit tunnel (NPET), in close proximity to the CCA end of the peptidyl-tRNA located in the ribosomal P site. Critically, the extended pyridine side chain of **KU13** at the C11 position interacts specifically with nucleotide U2847 in the NPET. This interaction induces a nearly 180° rotation of U2847, enabling π–π stacking between the pyridine ring of **KU13** and U2847. A second π–π stacking interaction is formed with adjacent nucleotide G2846, effectively sandwiching the **KU13** molecule between the two ribosomal bases. Additionally, the **KU13** benzene ring establishes further π–π stacking with U2016. These cooperative non-covalent interactions result in a structurally driven anchoring of **KU13** within the ribosomal tunnel, a mechanism that appears distinct from that of AZM and other analogues lacking the C11 side chain. This unique binding mode likely contributes to the superior bactericidal activity of **KU13** and its ability to overcome macrolide resistance, which is often mediated by alterations in the ribosomal binding site. Thus, the structural insights provided by cryo-EM strongly support the therapeutic potential of KU13 and the broader applicability of BMAD-driven modifications in antibiotic development.

The work of Isozaki et al. paves the way for the diversification of complex bioactive molecules, including glycosides and antibiotics, through a novel boron-mediated catalytic pathway [66]. This BMAD methodology enables efficient, regio- and stereoselective glycosylation of unprotected substrates, significantly reducing the need for protecting group strategies and aligning well with principles of green chemistry. Its compatibility with downstream functionalization techniques such as cycloadditions and SPAAC reactions further broadens its utility for applications in medicinal chemistry and chemical biology. As demonstrated in the selective labeling of glycosides and the rational enhancement of antibiotic activity, BMAD stands as a powerful tool for late-stage molecular editing—offering new opportunities for drug discovery, development, and the mechanistic interrogation of bioactive small molecules.

## 6. Enzyme-Independent Prodrug Activation Mechanism by Boron-Based Compounds

**Figure 15:**
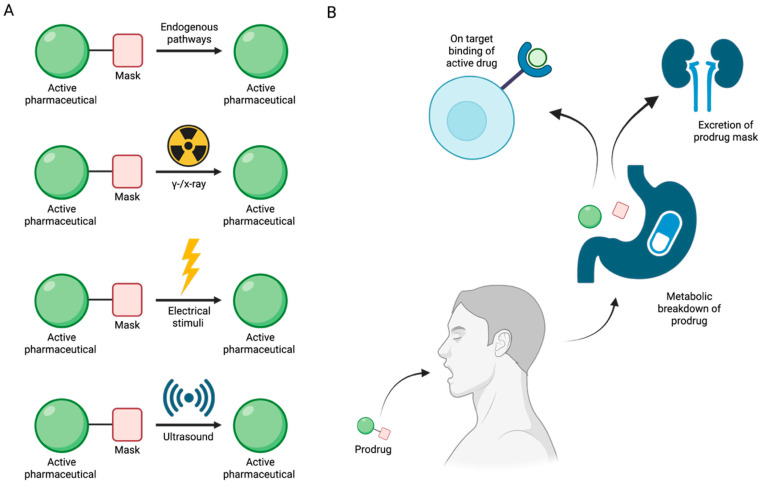
Overview of prodrugs and their activation methods. (**A**) Various activation mechanisms of prodrugs. Endogenous activation pathways involve metabolic processes such as enzymatic cleavage and pH-sensitive responses. External stimuli including λ- and x-ray irradiation, localized electrical fields, and ultrasound are non-endogenous methods of prodrug activation. (**B**) General concept of oral prodrug use. A bioinert formulation of a pharmaceutical is taken orally to increase overall bioavailability and downregulate off-target effects. The prodrug is designed to breakdown into the active pharmaceutical through metabolic pathways or external stimuli, at which point the active drug component undergoes on-target binding, while the prodrug mask is excreted. Figures are created using BioRender. Created in BioRender. Frooman, M. (2025) https://BioRender.com/efdaj0n.

The limited stability of many pharmaceuticals prior to reaching their intended biological targets has necessitated the development of prodrugs, bioinert, easily absorbed compounds designed to undergo controlled bioconversion into active therapeutics [71]. This conversion can occur via endogenous biological pathways involving physiological phenomena such as pH changes [72] and elevated levels of reactive oxygen species [73] or be externally triggered by gamma/X-ray photoactivation [74], electrical stimuli [75], or ultrasound [76] (Figure 15A). By regulating the timing and location of prodrug activation, it is possible to minimize systemic toxicity and premature metabolic degradation of the active drug [71] (Figure 15B).

**Figure 16:**
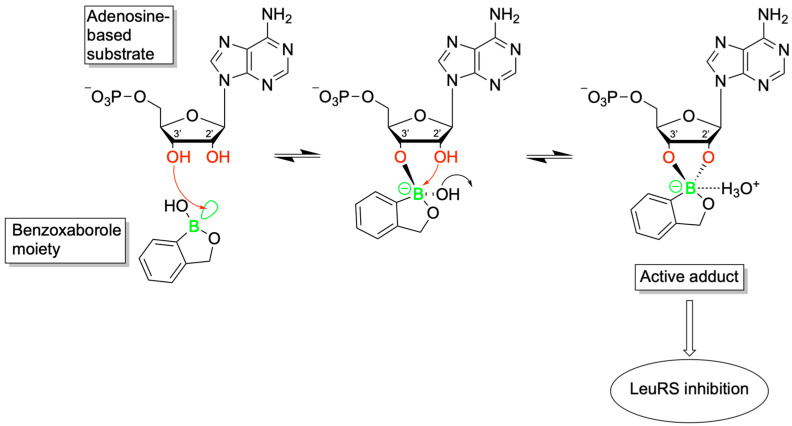
Scheme depicting the enzyme-independent cyclization between adenosine and a representative benzoxaborole prodrug. The ribose 2′ and 3′ hydroxyl groups of an adenosine activator attack the electrophilic boron center of the benzoxaborole moiety, producing the activated version of the drug with a negatively charged tetrahedral boron center. This active adduct is capable of inhibiting the bacterial LeuRS enzyme. Figures are created using ChemDraw.

Benzoxaboroles represent a promising class of drug scaffolds with activity against bacteria [77,78], protozoa [79], and fungus [80], as well as uses in treating atopic dermatitis [81]. In a recent study, Hoffmann et al. [82] elucidated the activation mechanism of a subset of benzoxaborole compounds targeting leucyl-tRNA synthetase (LeuRS), an enzyme that plays a crucial role in protein biosynthesis [83]. This study provides the first biophysical and structural evidence supporting the long-standing hypothesis that benzoxaboroles inhibit LeuRS by covalently modifying its editing domain through an adenosine-dependent mechanism. The oxaborole moiety plays a central role in this mechanism by undergoing enzyme-independent covalent cyclization with the ribose hydroxyl groups of adenosine-containing molecules such as ATP, AMP, or the terminal adenosine of tRNALeu (Figure 16). This reaction forms a reversible covalent adduct, facilitating LeuRS inhibition through site-specific interaction [82].

**Figure 17:**
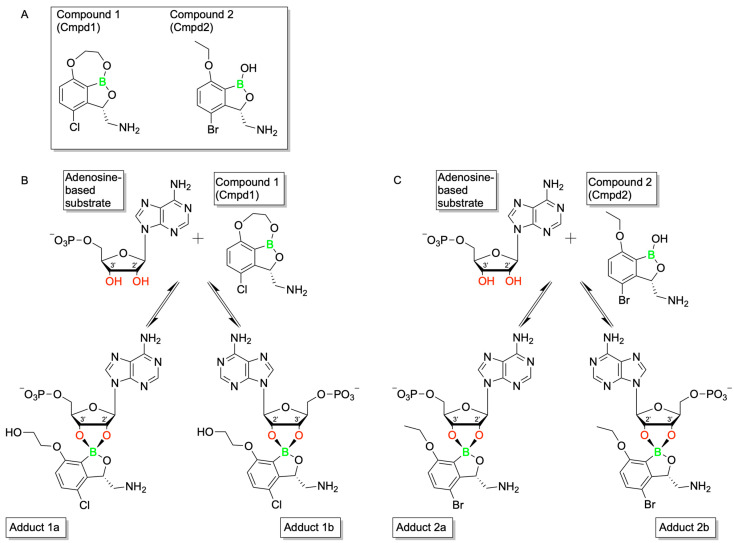
Benzoxaboroles complex with the 2′ and 3′ hydroxyl groups of adenosine riboses. (**A**) Benzoxaborole prodrugs Compound 1 (Cmpd1) and Compound 2 (Cmpd2). The boron centers are colored green. (**B**) Inhibition adducts formed by Cmpd1 with adenosine-containing molecules. Through sequential association of the 2′ and 3′ ribose hydroxyls with Cmpd1, two diastereomers, Adducts 1a and 1b, are formed. Ribose hydroxyls are colored red. Type “a” and “b” adducts are labelled based on the relative position of 2′ and 3′ ribose hydroxyls (**C**) Inhibition adducts formed by Cmpd2 with adenosine-containing molecules. Through sequential association of the 2′ and 3′ ribose hydroxyls with Cmpd2, two diastereomers, adducts 2a and 2b, are formed. Ribose hydroxyls are colored red. Type “a” and “b” adducts labelled based on the relative position of 2′ and 3′ ribose hydroxyls. Figures are created using ChemDraw.

To characterize this inhibition, Hoffmann et al. employed isothermal titration calorimetry (ITC) using recombinant *M. tuberculosis* LeuRS and two benzoxaborole compounds (Cmpd1 and Cmpd2) (Figure 17A) [82]. In the absence of adenosine-based cofactors, no binding was observed in the LeuRS binding pocket, indicating that proper benzoxaborole activation had not been achieved. In contrast, strong binding to the LeuRS editing site was observed in the presence of AMP or related adenosine-containing molecules. Cmpd1 emerged as the most potent in vitro LeuRS inhibitor reported to date, likely due to favorable polar interactions within the LeuRS binding pocket that lower binding enthalpy. Subsequent ITC and NMR analyses confirmed that this inhibition mechanism is consistent across multiple adenosine-based activators, including tRNALeu, and resulted in similar binding affinities across all Cmpd1-containing adducts. Both Cmpd1 and Cmpd2 formed two diastereomers through conversion of the electrophilic sp^2^ boron center to a tetrahedral, negatively charged sp^3^ configuration upon complexation with an adenosine-containing activator (Figure 17B,C). These mechanistic findings support the classification of benzoxaboroles as a new class of adenosine-dependent, boron-based prodrugs.

**Figure 18:**
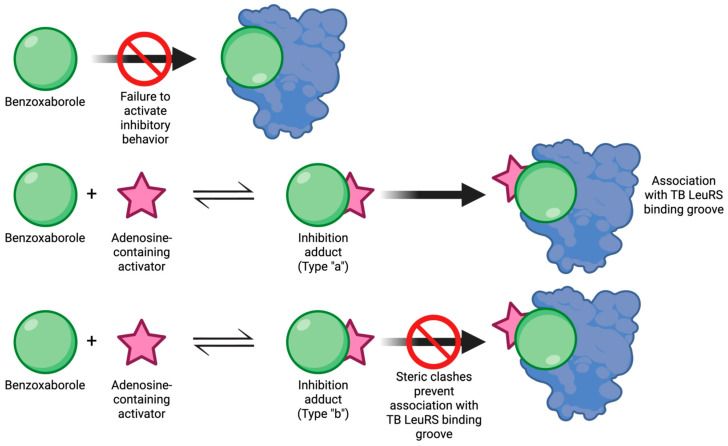
Benzoxaborole prodrugs are activated through covalent association with adenosine-containing activator molecules, which results in type “a” and “b” diastereomers. Type “a” adducts successfully associate with the TB LeuRS binding groove, while type “b” adducts fail to bind due to steric clashes. Without these activators, inhibitory behavior is not displayed. Created in BioRender. Frooman, M. (2025) https://BioRender.com/5y59ofw.

Crystallographic studies of benzoxaborole/AMP complexes in the presence of TB LeuRS confirmed direct binding at the LeuRS editing site. Only one diastereomer (type “a”) was observed in crystal structures, with in silico modeling revealing steric clashes that likely prevent the alternative (type “b”) adduct from binding (Figure 18). Additionally, activation of Cmpd1 led to cleavage of its pseudo-dioxepane moiety, yielding an oxyethanol group critical for high-affinity binding to the LeuRS site. This interaction results in stronger binding compared to Cmpd2, as corroborated by ITC measurements. Importantly, this enhanced binding translated to improved in vivo efficacy as demonstrated by Palencia et al. [84].

Further study through 1H-1H NOESY experiments revealed that the covalent adduct formation between benzoxaboroles and adenosine derivatives is fully reversible on a timescale of seconds, with interconversion between diastereomers occurring via hydrolysis of either B–O bond. Water molecules, present in the LeuRS active site, play a critical role in stabilizing these interactions. Structural data indicate that the water molecule exists in a hydroxonium-like state, allowing for a favorable electrostatic interaction with the negatively charged oxaborole group. Interestingly, pH-dependent binding studies showed that stable complex formation between benzoxaboroles, adenosine-based cofactors, and LeuRS occurs only under near-physiological conditions. Binding affinity decreases significantly at more alkaline pH values, indicating that protonation states play a critical role in maintaining the integrity of the inhibitory adduct.

The stabilizing electrostatic interaction of the hydroxonium ion with the negatively charged boron atom of the benzoxaborole compounds further helps to explain the mechanism which yields the preferred “a” type diastereomers (Figure 2). Briefly, ribose hydroxyl 3′ likely performs nucleophilic attack on the boron center, followed by cyclization by hydroxyl 2′, which in turn pushes out the stabilizing hydroxonium ion. Notably, this proposed mechanism is conserved across multiple pathogenic LeuRS sites across both eukaryotic and prokaryotic species.

To deduce whether Cmpd1 and Cmpd2 functionality was specific to human ATP, AMP, and tRNALeu, ribose-based biomolecules from humans and bacteria were tested at similar concentrations to that of physiological ATP (5–10 mM). While NMR and ITC experiments confirmed that the covalent adduct formation of Cmpd1 with other biomolecules was possible, when each adduct was titrated into a solution of TB LeuRS, no binding to TB LeuRS was observed. This experiment highlighted the necessity of the complete adenosine moiety to enhance the binding conditions of benzoxaborole/TB LeuRS complexes.

This study by Hoffmann et al. provides the first detailed structural and biophysical evidence of the route by which benzoxaborole-based prodrugs achieve adenosine-dependent selective inhibition of LeuRS [82]. Ribose hydroxyl positioning and pH-sensitive interactions, which mediate the binding of benzoxaborole adducts in the binding groove of LeuRS, enable conserved pathways of potent inhibition across diverse LeuRS sites. Together, these findings establish benzoxaboroles as a promising class of boron-based prodrugs with broad therapeutic potential, opening new avenues for the rational design of targeted, bioresponsive inhibitors.

## 7. Novel Benzoxaborole for the Treatment of Visceral Leishmaniasis

Visceral leishmaniasis (VL) is a neglected vector-borne tropical disease that targets major organs, including the liver, skin, lymph nodes, bone marrow, and spleen [85]. While many infections remain latent until immunosuppression triggers clinical manifestation, untreated symptomatic VL almost invariably results in death, either directly from the disease itself or indirectly via hemorrhaging, secondary infection, or concomitant pathologies. With transmission closely linked to poor socioeconomic conditions and high prevalence in densely populated urban areas throughout the world, there is a critical global need for safe, affordable, and accessible treatments [85].

**Figure 19:**
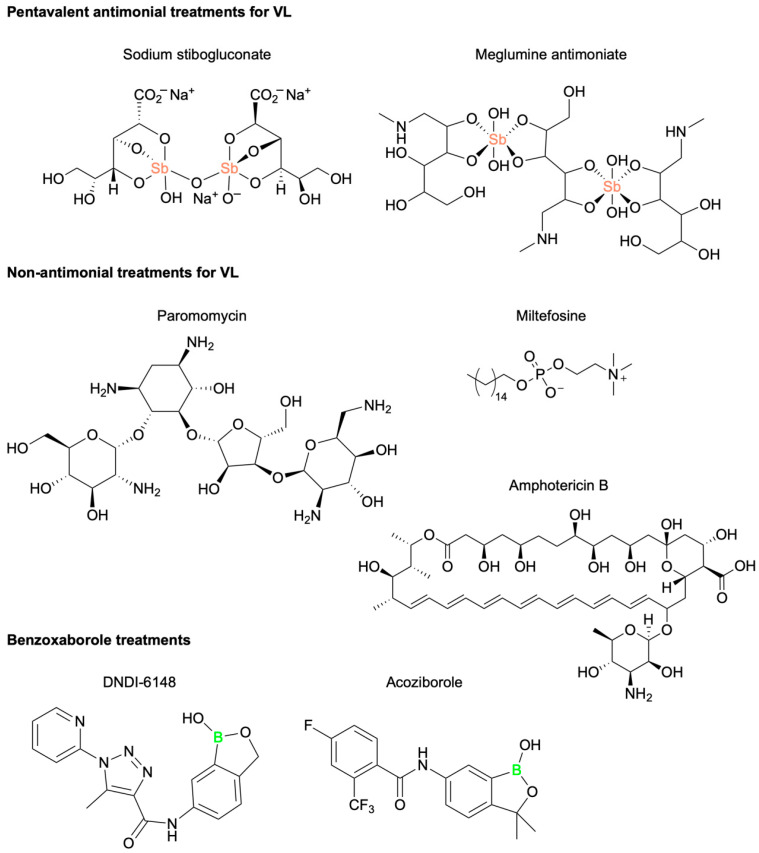
Structures of active compounds in current VL treatments. Pentavalent antimonial therapies, including sodium stibogluconate and meglumine antimoniate, are shown at the top. Non-antimonial treatments, paromomycin, miltefosine, and amphotericin B are shown in the middle. VL benzoxaborole scaffold drug candidate DNDI-6148 and the related anti-HAT drug, acoziborole, shown on the bottom. Antimony is depicted in pink, and boron is depicted in green. Figures are created using ChemDraw.

VL treatment strategies are influenced by various factors, including the *Leishmania* parasite species involved, geographic location, and the presence of co-morbid conditions. Chemotherapeutic interventions, though often effective, are limited by significant drawbacks, including high cost, parenteral administration, and physical burden on the patient [86]. The primary VL-mediating pharmaceutical agents in clinical use currently include pentavalent antimonial (Sb^v^) compounds, sodium stibogluconate (SSG), and meglumine antimoniate (MA), as well as non-antimonial compounds paromomycin (PM), miltefosine (MF), and amphotericin B (AmpB) (Figure 19) [87]. In recent decades, the clinical efficacy of these pentavalent antimonial drugs has significantly declined [88,89]. Reports dating back to the 1980s by Thakur et al. have detailed the rising frequency of treatment failures associated with these first-line defenses [90]. In 1999, Lira et al. demonstrated that *Leishmani Donovani* isolates from infected patients with reported Sb^v^ resistance required five times the standard dose of SSG to achieve a comparable in vitro efficacy [91]. This elevated dosing introduces a heightened risk of severe side effects, including renal toxicity, nephrotoxicity, cardiotoxicity, and death [92]. Additionally, concerns related to the non-antimonial drugs’ teratogenicity, injectable administration, cold storage conditions, or financial burden underscore the urgent need for a non-antimonial, non-invasive alternative for VL therapy.

Within the past decade, at least five novel therapeutic candidates for the treatment of VL have entered Phase I clinical trials [93,94]. Among these, DNDI-6148, a benzoxaborole scaffold drug developed by Mowbray et al., demonstrated potent in vitro and in vivo potency against two *Leishmania* parasite species, *L. infantum* and *L. donovani,* in hamster models (Figure 19) [95]. A ten-day in vivo treatment with DNDI-6148 resulted in near-complete parasitic clearance from all tested organs. The compound exhibited a favorable pharmacokinetic profile, and the oral formulation achieved a low ED50. DNDI-6148 completed Phase I clinical trials in 2022, showing good tolerability and promising efficacy, but further development of this pharmaceutical candidate has since been deprioritized due to concerns related to reproductive toxicity and teratogenic effects in pregnant patients [95]. However, to reduce the toxicity of DNDI-6148 through structural modifications, the primary focus is on addressing the pre-clinical reproductive toxicity observed in non-rodent species. The aim is to enhance selectivity for the parasitic CPSF3 enzyme over its human counterpart, thereby minimizing off-target effects that could be associated with toxicity. Key strategies include modifying the amide substituent to decrease lipophilicity and potentially altering its interaction with host cell targets, as well as enhancing interactions with the *Leishmania* CPSF3 binding pocket for improved selectivity [95].

**Figure 20:**
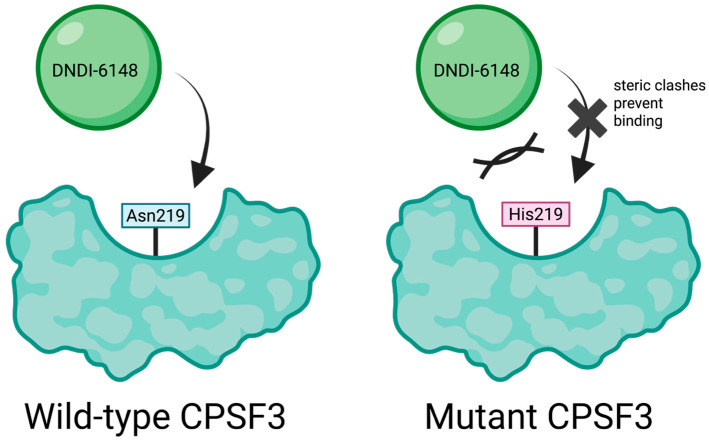
Cartoon depicting the binding of DNDI-6148 to the CPSF3 enzyme in *L. donovani*. Successful binding of DNDI-6148 to wild-type CPSF3 containing an Asn219 residue is shown on the left. Inhibited binding of DNDI-6148 to mutant CPSF3 containing a His219 mutation is shown on the right. DNDI-6148 is depicted as a green sphere. The CPSF3 binding groove is simplified with only position 219 visible. Created in BioRender. Frooman, M. (2025) https://BioRender.com/kydac6b.

Despite this clinical hold, the benzoxaborole scaffold remains a promising chemical framework for anti-parasitic drug development. DNDI-6148 shares several structural similarities with acoziborole, an approved clinical treatment for human African trypanosomiasis (HAT) [96] (Figure 19). Previous mode of action (MoA) studies with acoziborole revealed that it targets the parasitic Cleavage and Polyadenylation Specificity Factor 3 (CPSF3), an essential endonuclease involved in regulating the translation of mRNA in *Trypanosoma brucei* [97] (Figure 19). Orthologs of CPSF3 have been identified as benzoxaborole drug targets in other parasitic organisms, including *Plasmodium falciparum* [98], *Toxoplasma gondii*, and *Cryptosporidium spp* [99]. Based on these findings, Mowbray and colleagues. hypothesized that DNDI-6148 may also target CPSF3 in *L. donovani*. To test this theory, the authors assessed the susceptibility of both wild-type and transgenic *L. donovani* expressing modified CPSF3 binding grooves (Asn219His) to DNDI-6148. The engineered parasite line exhibited a 3.6-fold reduction in susceptibility to DNDI-6148, strongly suggesting that CPSF3 is the principal target for this anti-leishmaniasis benzoxaborole drug (Figure 20).

To further characterize the molecular interactions between DNDI-6148 and the target *L. donovani* CPSF3, a homology model was constructed based on the bacterial homologue, *Thermus thermophilus* TTHA0252 (PDB ID: 3IEM)^19^, which shares 30% sequence identity with the parasite enzyme. The model revealed that DNDI-6148 interacts with an activated zinc-water complex coordinated by conserved histidine and aspartic acid residues within the catalytic site of CPSF3. Furthermore, the amide moiety in position 6 of the benzoxaborole scaffold directs the pyridyl-triazole substituent of DNDI-6148 to initiate a pi-stacking interaction with Tyr 370 of the CPSF3 binding groove, further stabilized by a hydrogen bonding interaction with Thr 218. While no direct interaction was observed between DNDI-6148 with Asn 219 in the wild-type enzyme, substitution with His at this position created steric clashes with the pyridyl-triazole group, disrupting optimal ligand binding. The proposed binding model aligns with that of acoziborole’s interaction with *T. brucei* CPSF3. This proposed binding model aligns with the interactions of acoziborole with T. brucei CPSF3. Notably, the human CPSF3 homolog contains a Tyr residue at the position of Asn219 in *L. donovani*, likely preventing DNDI-6148 from binding human CPSF3, thereby reducing the risk of off-target toxicity.

The limitations of current VL treatments underscore the urgent need for novel, orally available therapeutics. Benzoxaborole-based compounds based on the structure of DNDI-6148 offer a promising alternative, with demonstrated potency against *Leishmania* species and a precise, parasite-specific mechanism of action via CPSF3 inhibition. Although development of DNDI-6148 has paused due to toxicity concerns, the structural insights and mechanistic understanding derived from its study provide a valuable framework for the rational design of next-generation antileishmanial agents. Continued exploration of the benzoxaborole scaffold, with a focus on reducing toxicity while maintaining efficacy, holds considerable promise for addressing this global health challenge [95].

## 8. Antifungal Activity of 3-Substituted-2(5H)-Oxaboroles

**Figure 21:**
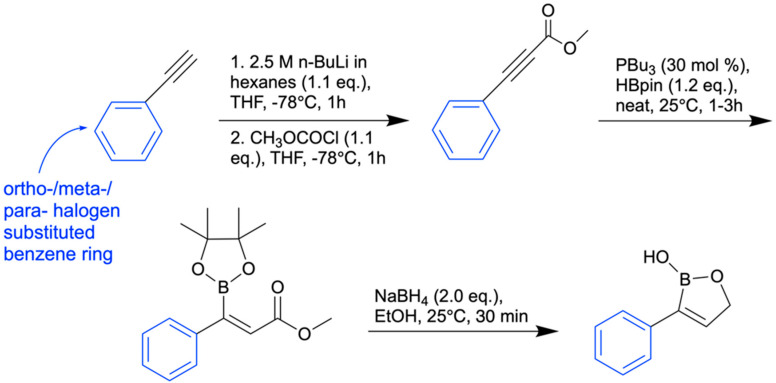
Comparison between benzoxaborole and 3-substituted-2(*5H*)-oxaborole. 3-substituted oxaboroles offer rotational freedom not found in benzoxaboroles, enabling more varied binding conformations within the protein active site. Small halogen substituents provide improved antifungal activity in both scaffolds. Figures are created using ChemDraw.

**Figure 22:**
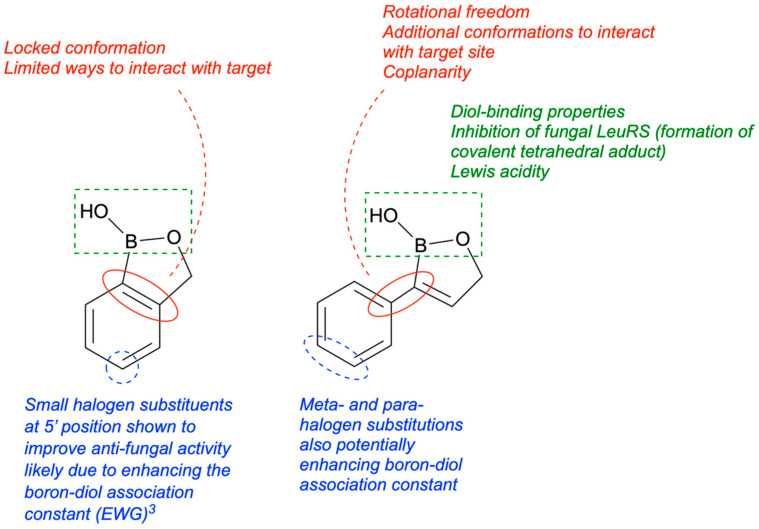
Synthesis scheme of 3-substituted-2(*5H*)-oxaboroles. Acetylenes with halogen substituted benzene rings are first treated with n-butyllithium and then methyl chloroformate to form alkynoates. This is followed by hydroboration with pinacolborane and tributylphosphine, and finally reduction with sodium borohydride to obtain the desired products. Figures are created using ChemDraw.

In 2024, Campbell and her team synthesized twenty-five 3-substituted-2(5H)-oxaboroles to examine their antimicrobial properties [100]. The importance of this subclass of oxaboroles lies in the potential for conformational flexibility due to bond rotation around the aryl and oxaborole rings, allowing boron to adopt different conformations as an electrophile. This differentiates them from benzoxaboroles like tavaborole and crisaborole. Benzoxaborole derivatives may show antibacterial, antiviral, or antifungal effects (such as tavaborole). Benzoxaboroles have low toxicity and greater acidity than PBA because of the transition from sp^2^ hybridization of the boron atom to sp^3^ when reacting with water, which releases strain and forms a tetrahedral adduct [101]. The synthesis of 3-substituted oxaboroles aims to enhance the antifungal properties of benzoxaborole derivatives by facilitating bond rotation, thereby improving interactions with enzyme active sites and preventing protein synthesis (Figure 21). The complete synthetic scheme for the synthesis of 3-substituted-2(5H)-oxaboroles is presented in (Figure 22).

Of the 25 oxaboroles, those with halogen atoms in the *meta* position were preferred over the para and ortho positions; the ortho-positioned halogen-containing oxaboroles were found to be ineffective. The 3-iodo-benzene substituted oxaborole showed the lowest minimum inhibitory concentration (6.25 µg/mL) against a fungal pathogen (*P. chrysogenum*). The team hypothesized that coplanarity of the aryl and oxaborole rings was a key factor in antimicrobial activity, and the *ortho*-substituted oxaborole’s lack of rotational freedom caused either steric clash when binding or inability to exhibit the preferred conformation. Generally, all bulky or methoxy substitutions to the benzene ring caused loss of growth inhibition, indicating the importance of coplanarity between the two rings.

**Figure 23:**
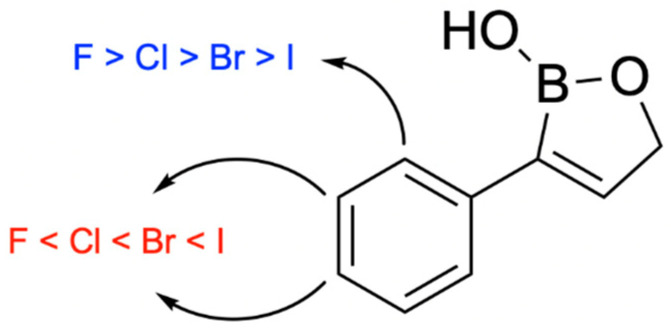
*Meta* and *para* located halogen substituted 3-oxaboroles show increased antifungal activity with increasing halogen size, while *ortho* substituted 3-oxaboroles show the opposite trend, likely due to steric clash preventing free rotation. Figures are created using ChemDraw.

For both meta and para halogen substitutions, the minimum inhibitory concentration decreased with increasing halogen atom size, resulting in a 4-fold increase in efficacy for iodine substitution compared to fluorine substitution (Figure 23). The synthesized 3-substituted oxaborole derivatives were generally ineffective against bacterial pathogens, save for mild growth inhibition of *E. coli*. None of the antimicrobial compounds (MIC ≤ 25 µg/mL against one or more pathogens, excluding *S. cerevisiae*) possessed hemolytic activity or cytotoxic effects towards mammal cells. In summary, halogen substitutions in the *meta* position were greatly preferred, and the synthesized 3-substituted oxaboroles were demonstrated to be nontoxic and nonhemolytic. Future studies will aim to understand the mechanism of action and identify fungal/yeast targets.

## 9. Boron-Containing Compounds for the Treatment of Alzheimer’s Disease (AD)

In 2018, Lu and team developed and evaluated a series of multi-target boron-containing compounds meant for Alzheimer’s disease (AD) treatment with the ability to function as β-amyloid (Aβ) inhibitors, metal-chelating agents, and antioxidants [102]. AD is a neurodegenerative disorder causing progressive memory loss and cognitive impairment, with Aβ [103] deposits being a key pathological marker [104]. Another hallmark feature of AD pathogenesis is free-radical and oxidative damage [105], making antioxidants a potential treatment to slow neuronal degeneration [106].

**Figure 24:**
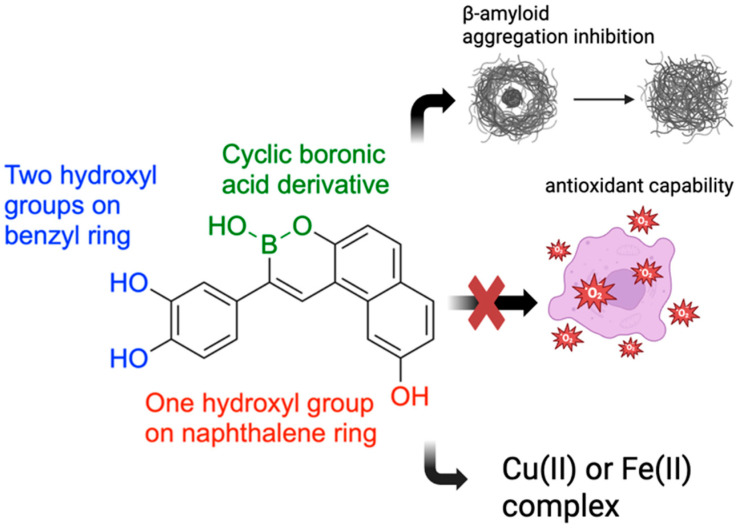
Structural and chemical properties of lead boron-containing compound (BCC). The model BCC was characterized by two hydroxyl groups on its benzyl ring, and one hydroxyl group on its naphthalene ring, which are likely due to their hydrogen bonding activities when interacting with the target proteins. This compound exhibited significant inhibition of Aβ aggregation and antioxidant capacity, as well as the ability to form complexes with copper(II) and iron(II). Figures are created using ChemDraw.

**Figure 25:**
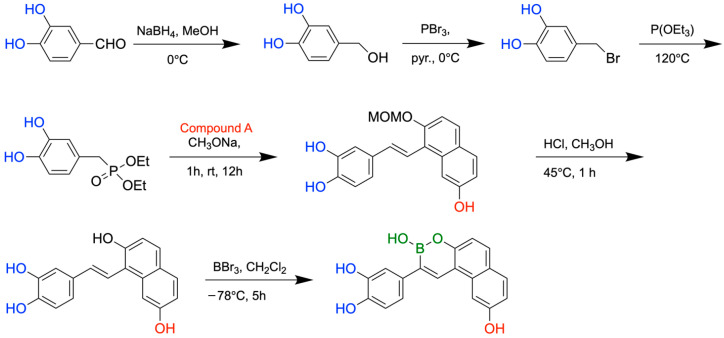
Synthesis scheme for lead boron-containing compound. The substituted benzaldehyde is treated with sodium borohydride in methanol to obtain an alcohol, which is then treated with PBr3 in the presence of pyridine to form a bromide. These bromides are converted into Wittig reagents and are used for Wittig olefination with previously mentioned compound A. The methoxymethyl group is then removed using hydrochloric acid, and the compound is finished through cyclization using boron tribromide. Figures are created using ChemDraw.

To evaluate inhibition of Aβ aggregation, the boron-containing compounds were evaluated using a thioflavin-T fluorescence binding assay. Nearly half of the boron-containing target compounds were found to be more potent than curcumin, a known inhibitor of amyloid aggregation used as the reference compound. It was shown that compounds with two hydroxyl groups on the benzyl ring and one hydroxyl group on the naphthalene ring were the most potent. Furthermore, the Removal of one hydroxyl group on each ring significantly lowered inhibition, supporting past studies that demonstrate polyphenol-protein interaction is heavily reliant on hydrogen bonding (Figure 24). Antioxidant activity was examined using the oxygen radical absorbance capacity assay. All synthesized boron-containing compounds displayed antioxidant capacity; however, the most potent molecule was again a compound containing two hydroxyl groups on the benzyl ring and one hydroxyl group on the naphthalene ring. Further testing of one of these compounds revealed a red shift in the maximum absorbance peak upon the addition of CuSO_4_ or FeSO_4_, indicating the formation of copper(II) and iron(II) complexes. In conclusion, the synthesized boron-containing compounds exhibited antioxidant, biometal chelator, and Aβ aggregation inhibitor properties, giving them potential as future structural scaffolds for the treatment of AD. The synthetic routes for the new boron-containing compounds as Aβ aggregation inhibitors are presented in Figure 25.

## 10. Boron in Biological Target Engagement

In 2017, Diaz and Yudin analyzed various boron inhibitors and their methods of covalent binding to describe boron’s mechanistic and structural properties, emphasizing its ‘chameleonic’ behaviour and unique coordination strategies [14]. Boron’s various methods of molecular target engagement make it potentially superior to other electrophiles that provide only one type of interaction.

In the active site of an enzyme, the covalent capture of boron enables it to transform from a trigonal-planar structure to an anionic tetrahedral structure, allowing the surrounding hydroxyl groups to accept or donate hydrogen bonds [107]. Through testing in aqueous alcohol solutions, it was demonstrated that these boron-bound hydroxyl ligands may also change during interaction, being switched out with alcohols or diols to form new hydrogen bonds [108]. Boron-based inhibitors with low stability may also be coordinated with groups such as *N*-methyliminodiacetate (MIDA), protecting boron from external nucleophiles and slowing hydrolysis [109]. This coordination has been shown to enhance the pharmacokinetic profile of boron-based inhibitors, suggesting potential as an improved drug-delivery method [110].

Cyclic boronates have restricted conformations, which can lead to a reduction in protein binding entropy. Cyclic boronates have been shown to have inhibitory properties towards human 20s proteasome and β-lactamase enzymes [111,112]. Enzymes may even react with the cyclic boronate in its closed form before hydrolysis at boron to trap it in its extended linear conformation [113]. This property was also seen in inhibitors with endocyclic B-N bonds, which are weaker than B-O bonds. Despite being weaker than B-O bonds, substitution of a hydroxyl ligand for the nitrogen atom of an amino side chain is observed, as in D-(*S*)-aminoboronic acid, an inhibitor of *γ*-chymotrypsin, creating a dicovalent complex with serine and histidine [114]. Tricovalent binding is also possible, as observed in an inhibitor for a penicillin-binding protein [115]. This phenomenon is not observed in proteins with nucleophilic thiolate side chains [116].

**Figure 26:**
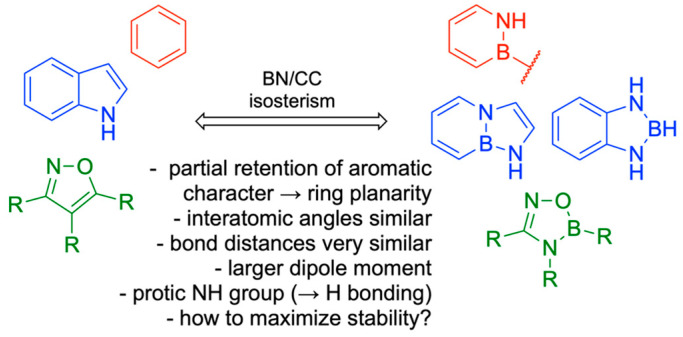
Examples of carbon-carbon to boron-nitrogen isosterism, along with some features retained and changed after substitution. Benzene and its azaborine equivalent are in red. Indole and two boron-nitrogen indole isosteres are depicted in blue. Isoxazole and oxadiazaborole are in green. These compounds are isoelectric and isostructural but may be subject to higher rates of degradation due to instability. Figures are created using ChemDraw.

Another feature of boron-based inhibitors is their ability to be modified with bulky groups, enabling them to bind in unique and unusual conformations within narrow protein pockets. For example, a benzyl group in a protease inhibitor can induce a trigonal covalent adduct instead of a tetrahedral one, demonstrating that boron can also form neutral tricoordinate adducts in proteins [117]. Isosteric replacement of a carbon-carbon bond with a boron-nitrogen bond can help keep boron trigonal within the active site while also improving hydrogen bonding and solubility [118]. For instance, replacing a C=C bond in benzene creates 1,2-azaborine, which retains partial aromaticity and a planar ring structure, while also making boron inaccessible to nucleophiles. 14 B-N isosteres have also been synthesized for other molecules like isoxazoles and indoles, maintaining similar bond distances and angles with enhanced antifungal activity [119] (Figure 26). Structure-activity relationship analysis on a serine/threonine kinase (*ROCK2*) inhibitor, benzoxaborole, showed that electron-withdrawing substitutions on the benzyl ring can lower the pKa and stabilize the sp2 configuration of the boron atom [120]. Using these modifications, developers can adjust the properties of potential medicinal boron-containing compounds and reduce their binding to external nucleophiles.

**Figure 27:**
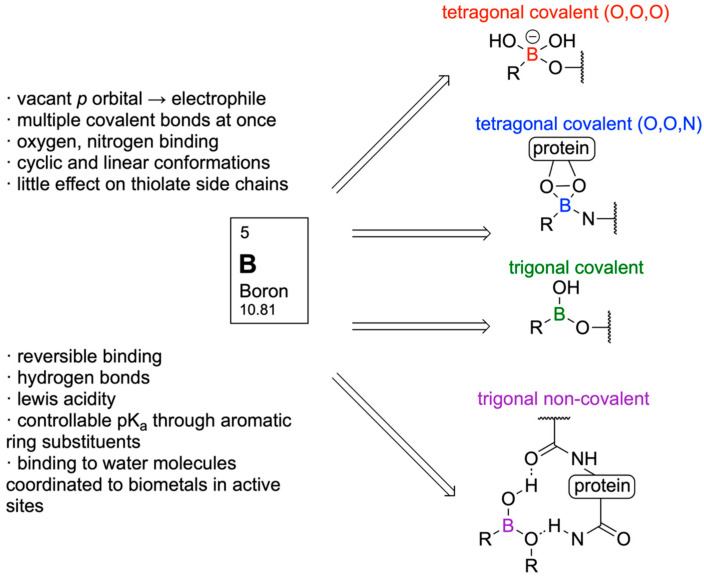
Boron-containing compounds (BCCs) may engage in four types of binding at the enzyme active site. Tetragonal covalent binding can be either oxygen or nitrogen bonds. Trigonal non-covalent interactions demonstrate how the boronic acid inhibitors may interact with protein side-chains. BCCs have various unique characteristics and binding modes. Figures are created using ChemDraw.

**Figure 28:**
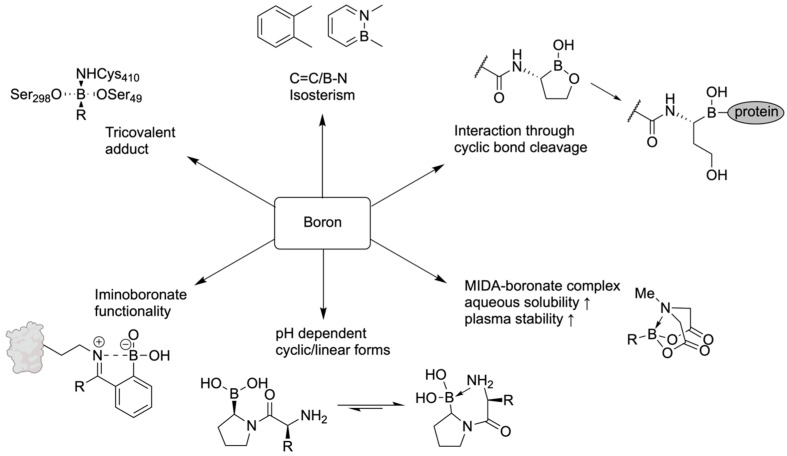
Summary of some lesser-known features and binding modes of boron-containing molecules in the biological context. Boronic acid derivatives can interact with proteins in a variety of ways, including through iminoboronate formation or a tricovalent adduct. Boron-nitrogen bonds also provide a potential isostere of carbon-carbon double bonds. Within the biological context, the boronic acids can equilibrate between cyclic and linear forms, or a cyclic boronate can interact covalently through bond cleavage. Additionally, aqueous solubility and plasma stability of boronic acids may be improved by complexing them with MIDA. Figures are created using ChemDraw.

Summary of binding modes of boron-containing compounds across different biological enzymatic sites (Figure 27) and some lesser-known features and binding modes of boron-containing molecules in the biological context (Figure 28).

## 11. Boron in New Drug Chemotypes: Pharmaceutical Potential of Hemiboronic Naphthoids

In 2023, Kazmi and team explored the pharmaceutical potential and chemical properties of hemiboronic naphthoids, benzoxazaborines, and benzodiazaborines, aiming to use the B-OH bond as an isosteric and isoelectronic replacement for the carbonyl group (C=O) [121]. The boranol moiety has potential for introducing additional hydrogen bonding and covalent bonding with protein targets, along with better aqueous solubility. Model compounds based on these two scaffolds were synthesized and screened for antimicrobial activity, along with DMPK assays to examine their drug-like properties. Basic physical properties such as acidity, stability, and binding with common cellular carbohydrates were also examined. Additionally, benzoxa- and benzodiazaborine isosteres of olaparib, crisaborole, and tavaborole were evaluated and compared.

To determine which scaffolds would be suitable as different isosteres, the physical properties of the benzoxazaborines and benzodiazaborines were evaluated. The benzoxazaborines had a lower pK_a_ of ~5.5 and lack aromatic character in their boron-containing ring. This makes them suitable as isosteres of benzoxaboroles, giving them the ability to form an anionic tetrahedral adduct in the enzyme active site under biological conditions. Conversely, the benzodiazaborine analogues had a much higher pK_a_ (>14), along with a partially aromatic boron-containing ring, making them more suitable as isosteres of hydroxyquinolines, naphthols, or phthalazinones. Both classes exhibited aqueous solubility and stability, with minimal interaction with common saccharides, demonstrating specificity and a lack of off-target interactions.

**Figure 29:**
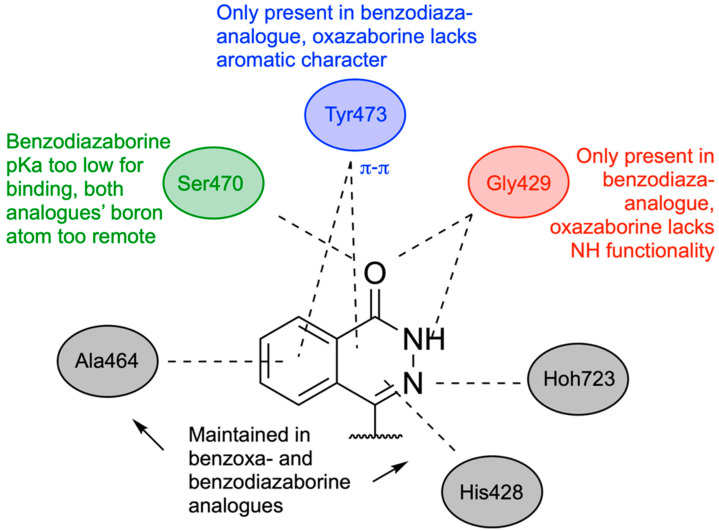
Protein-ligand interaction map of olaparib and the interactions affected by changing to boron-based isosteres. Ser470, which is usually involved in H bonding, loses this interaction when the carbonyl moiety is replaced with a boranol (B-OH) moiety due to increased distance from the residue. The benzoxazaborine analogue lacks two other interactions. One is the π-π interaction with Tyr473, which requires at least partial aromatic character in the boron-containing ring, such as in benzodiazaborine. The other is hydrogen bonding with Gly429, which is lost in the oxazaborine due to the lack of NH functionality. Figures are created using ChemDraw.

Benzoxaza- and benzodiazaborine isosteres of olaparib, a PARP inhibitor used in the treatment of ovarian cancer [122], were synthesized. The benzodiazaborine analogue exhibited superior PARP2 inhibition compared to the benzoxazaborine (0.12 µM vs. >1000 nM), likely due to the retention of aromatic character, allowing for π-π interactions to be preserved partially [123]. Additionally, the benzoxazaborine loses hydrogen bonding with Gly429 due to the loss of NH functionality compared to olaparib. Additionally, although the more acidic oxazaborine was predicted to interact covalently with Ser470, both drug isosteres likely placed boron too far away from Ser470′s hydroxyl group for binding (Figure 29).

Isosteres of crisaborole, a phosphodiesterase (PDE) inhibitor used for the treatment of atopic dermatitis, were also developed. Crisaborole mimics the tetrahedral intermediate in phosphodiesterase hydrolysis [124], making the benzoxazaborine heterocycle an expected suitable replacement scaffold due to its Lewis acidity being sufficient for forming an anionic tetrahedral adduct under biological conditions. Additionally, the heteroatoms of the boron-containing ring may all contribute to improved hydrogen bonding in the active site. The benzoxazaborine, as predicted, was superior to the benzodiazaborine analogue; however, both isosteres lacked significant inhibition of PDE. It is likely that the newly introduced 6-member heterocycles were too sterically demanding and unable to fit correctly in the binding pocket of PDE.

**Figure 30:**
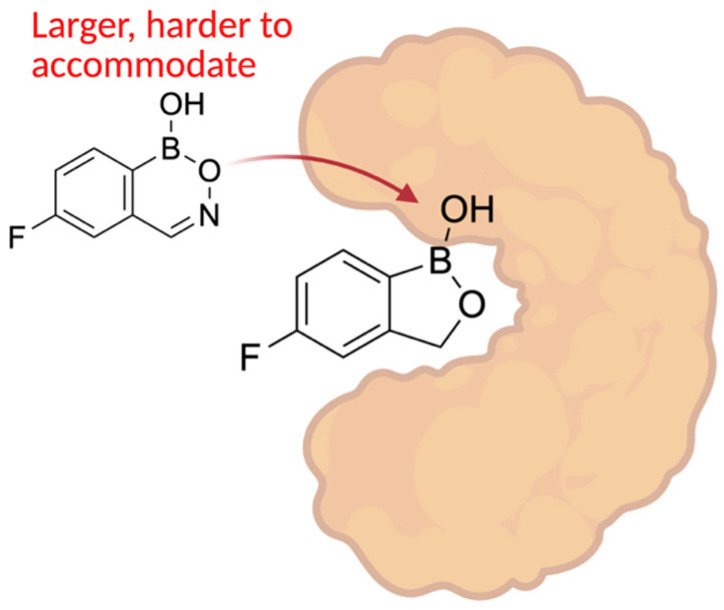
Tavaborole (MIC = 1 µM) vs. one analogue (MIC = 8 µM), showing the limited space in the enzyme binding pocket of fungal leucyl-tRNA synthetase. The additional N atom may also cause destabilizing electronic interactions, further decreasing activity. Figures are created using ChemDraw.

Finally, isosteres of the antifungal agent tavaborole were synthesized. Tavaborole functions by blocking the editing site of fungal leucyl-tRNA synthetase through the formation of a covalent tetrahedral adduct between boron and the tRNA 3’ terminal adenosine [125]. Similarly to crisaborole, benzoxazaborine’s boron and its ability to become tetracoordinate at biological pH made it a more suitable isostere than benzodiazaborine. The benzoxazaborine analogues displayed better antifungal activity than the benzodiazaborines (MIC = 8–16 µM vs. MIC >128 µM); however, none of the analogues were equal or superior to tavaborole. This is likely due to either destabilizing interactions with the nitrogen atoms introduced in the boron-containing ring or the steric demand of a 6-membered heterocycle in the binding pocket (Figure 30).

To examine the reaction potential and cross-reactivity of the benzoxa- and benzodiazaborine scaffolds, various reactions used in drug discovery were attempted. Late-stage amidation, Chan–Lam coupling, and Suzuki–Miyaura cross-coupling were all successful, yielding modest to high yields; however, the benzoxazaborines provided lower yields due to their inherent acidity. A small library of functionalized benzoborine derivatives was synthesized, and a select few were screened for antimicrobial activity. When tested against *S. aureus*, benzoxazaborine structures exhibited significantly lower minimum inhibitory concentrations than benzodiazaborine analogues, due to their acidity and ability to interact as a tetrahedral adduct at physiological pH. Screening against *C. albicans* demonstrated the same phenomenon, highlighting the importance of boron, which utilizes its tetracoordinate binding mode. In both *S. aureus* and *C. albicans* testing, fluorinated analogues of tavaborole were more active than their nonfluorinated counterparts, supporting the use of these heterocycles as isosteres of benzoxaboroles. DMPK assays showed high membrane permeability, moderate to high metabolic stability, and active membrane influx for benzoxazaborines.

## 12. Phenyl Boronic Acid (PBA)-Based Non-β-Lactam β-Lactamase Inhibitors Against KPC-2-Producers

Carbapenems are broad-spectrum *β-lactam* antimicrobials that inhibit bacterial cell-wall synthesis. Due to their resistance to most β-lactamases, such as extended-spectrum β-lactamases, they are reliable for use in severe infections. However, carbapenem-resistant bacteria that produce KPC-2 carbapenemases threaten to compromise the effectiveness of these last-resort antibiotics. KPC-2 is a *β*-lactamase capable of hydrolyzing all groups of *β*-lactam antibiotics, and some of their *β*-lactam-based *β*-lactamase inhibitors [126].

In 2025, Singh and team synthesized phenylboronic acid (PBA) isosteres and analogues that target KPC-2 [127]. Carboxylate-substituted phenylboronic acids were previously found to be KPC-2 inhibitors [128], and the replacement of the carboxylate group with a phosphonate group improved the inhibitory activity [129]. Accordingly, ten phosphonates and eight carboxylates were synthesized and tested.

The addition of a 2-propenylcarboxylate or 2-propanylcarboxylate group greatly improved inhibitory activity compared to PBA. Replacement of the carboxylate group with/an amide, methylsulfonyl, or sulfonamidomethyl group caused loss of almost all activity. Replacement of the carboxylate with a phosphonate group maintained inhibitory activity. Unsaturated carboxylates were slightly more active than unsaturated carboxylates, with 5-methoxy-substitution providing the most active carboxylate-based analogue (K_i_ = 0.095 μm). The most potent inhibitor among the phosphonate analogues had a meta-substituted fluorine (K_i_ = 0.07 μm).

**Figure 31:**
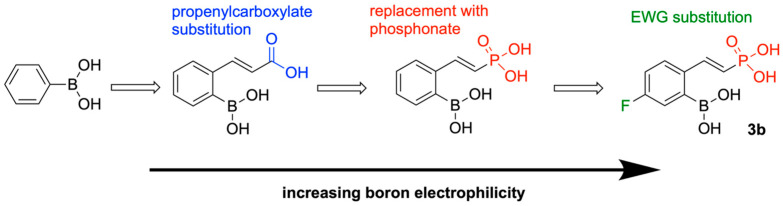
Stepwise changes to the phenylboronic acid scaffold causing increased boron electrophilicity. Previous studies demonstrated the KPC-2 inhibitory activity of carboxylate-substituted phenylboronic acids, followed by other research demonstrating that replacement of the carboxylate group with phosphonate led to increased inhibition. Singh and team discovered potent KPC-2 inhibitors by increasing the electrophilicity (and thus activity) of the boron atom through electron-withdrawing group substitution on the ring. Figures are created using ChemDraw.

**Figure 32:**
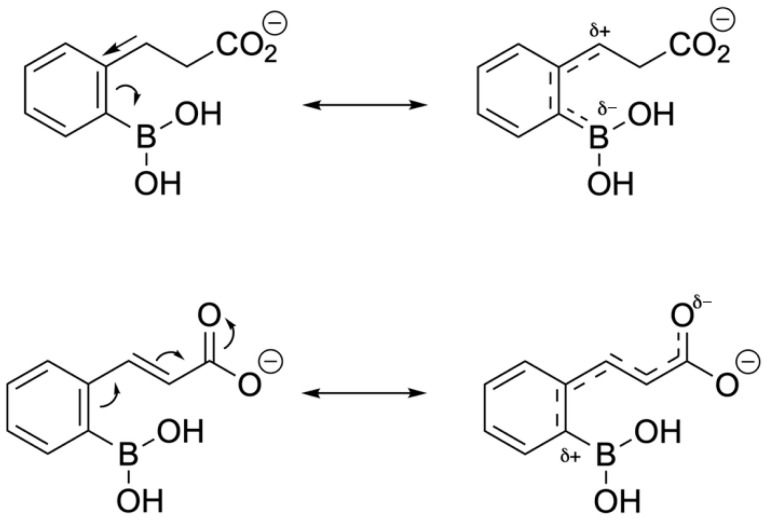
Electron distribution propanyl- and propenylcarboxylate substituted phenylboronic acids. The saturated propan-3-ylcarboxylate is electron-donating and increases the negative charge on the boron atom. Conversely, the unsaturated propen-3-ylcarboxylate functions as an electron-withdrawing group, providing a partial positive charge to the boron atom and making it more electrophilic and reactive. Figures are created using ChemDraw.

Through a nitrocefin hydrolysis assay, it was determined that the unsaturated phosphonate and carboxylate analogues were slow-reversible inhibitors, whilst the saturated carboxylates exhibited rapid reversibility/dissociation. The differing stoichiometric properties of the enzyme-inhibitor complex can be explained through electron distribution within the side chain and ring. The saturated side chain induces a negative partial charge on the boron atom, while the unsaturated side chain creates a positive partial charge. The electron-withdrawing quality of the propen-3-ylcarboxylate renders the boron atom more electrophilic and stabilizes the bonding with the nucleophilic serine residue in the active site. Similarly, electron-withdrawing substituents prolonged residence time, and electron-donating groups (i.e., methyl) decreased residence time (Figure 31 and Figure 32).

Synthesized PBA analogues displayed enhanced specificity toward KPC-2, and similarly to vaborbactam, the compounds were non-inhibitory when tested against bacteria alone. A combination of one PBA analogue (**3b**) with Meropenem, a carbapenem antibiotic, significantly increased bactericidal activity. Moreover, when compared to Meropenem-vaborbactam, which allowed for growth recovery of K. pneumoniae K47-25 after 24 h, the Meropenem-**3b** combination sustained suppression of bacterial growth. When evaluated in vivo against Meropenem monotherapy, Meropenem-**3b** significantly reduced bacterial burden in the lungs of mice infected with *K. pneumoniae* K47-25. This indicates that **3b** can potentiate Meropenem’s antibacterial activity and reverse carbapenem resistance.

## 13. Discovery and Optimization of a Novel Boronic-Acid (BA) Arginase Inhibitor

**Figure 33:**
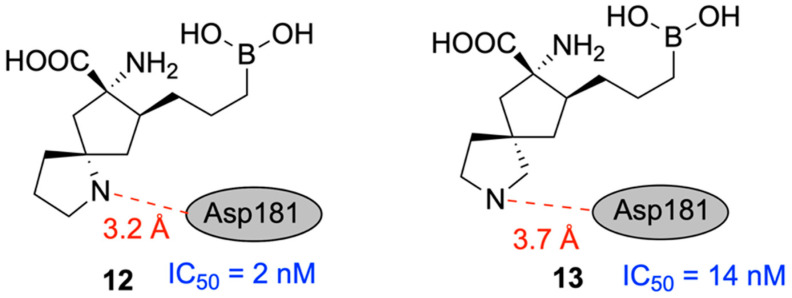
Compound **12** vs. compound **13.** The difference in arginase inhibition potency between the two compounds is theorized to be due to the distance of the electrostatic interaction between the secondary nitrogen and an oxygen on Asp181. Figures are created using ChemDraw.

In 2020, Mitcheltree and team designed novel inhibitors of human arginase based on fused 5,5-bicyclic ring scaffolds [130]. Arginase is a metalloenzyme shown to suppress T-cell activation and support tumor growth, making it a potential target for improving cancer immunotherapy [131]. Beginning with the hArg1 substrate mimetic ABH and two of its derivatives, which interact with the acidic residue Asp181 through hydrogen bonds, a new molecule, 3, was designed with a rigid bicyclic appendage and confirmed to interact with Asp181 electrostatically, resulting in improved inhibitory activity of hArg1 (IC_50_ = 29 nM). Potency was further increased in two different bicyclic scaffolds, **12** (IC_50_ = 2 nM) and **13** (IC_50_ = 14 nM) (Figure 33). The differentiated potencies in these two scaffolds were likely due to the varying nitrogen–oxygen distances between the compounds’ secondary nitrogen atoms and Asp181. The importance of this electrostatic interaction was confirmed through comparison with neutral analogues, which exhibited extremely low potency.

**Figure 34:**
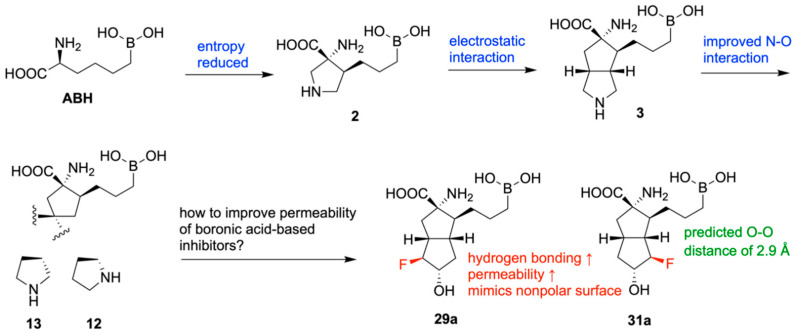
Design pipeline from (*S*)-2-amino-6-boronohexanoic acid (ABH) to derivatives **2, 3, 12, 13, 29a** and **31a** demonstrating increasing potency and permeability. Compound **2** introduces an anti-configuration, reducing conformational entropy through ring constraint. Compound **3** introduces a rigid bicyclic structure, allowing for stronger electrostatic interaction rather than hydrogen bonding. Compounds **12** and **13** significantly enhance the N-O interaction by reducing the distance. **29a** and **31a** features improved bioavailability when compared to **12** and **13** (11~16% F vs. 3~4%). This can be attributed to fluorine’s ability to improve the hydrogen bond donation of vicinal alcohols. Figures are created using ChemDraw.

The boronic acid moiety present in all investigated compounds is required for inhibiting hArg1, but unfortunately, it leads to low permeability. A variety of substituted derivatives were designed and tested; however, only **29a** (IC_50_ = 14 nM) and **31a** (IC_50_ = 37 nM) demonstrated both retained potency and improved bioavailability. This is attributable to fluorine’s ability to enhance the hydrogen bond donor strength of vicinal alcohols, providing stronger interaction with Asp181, along with mimicking the nonpolar surface of its unsubstituted analogue (Figure 34).

## 14. Boron in the Treatment of Chagas Disease

Chagas disease, caused by *Trypanosoma cruzi*, is a significant contributor to infectious myocarditis [132] and is responsible for approximately 10,000 deaths annually, according to the World Health Organization (WHO). While two current drugs (benznidazole and nifurtimox) are available, their practicality is limited by inconsistent efficacy and frequent side effects [133]. Benzoxaboroles, a group of boron-containing heterocyclic molecules, have demonstrated potency against pathogenic protozoans, fungi, and bacteria [98]. Here, Padilla et al. [133], developed and optimized a class of highly effective benzoxaboroles with high cure rates and specificity against *T. cruzi*. Among the candidates, AN15368 was identified as the most promising prodrug due to its consistency and excellent efficacy against T. cruzi in both in vitro and in vivo studies. Further testing in non-human primates (NHP) suggested a lack of acute toxicity or long-term consequences. Now, AN15368 is awaiting clinical trials, with the potential to save thousands of lives in the near future.

**Figure 35:**
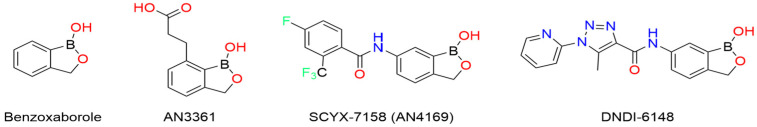
Structures of benzoxaborole and its derivatives: AN3661, SCYX-7158 (AN4169) and DNDI-6148. AN3661 combats *Plasmodium falciparum*, the most lethal malaria-inducing protozoan in humans. AN3661 is active in vitro and in mouse models by targeting cleavage and polyadenylation specificity factor subunit 3 (CPSF3) in *P. falciparumin* (PfCPSF3). SCYX-7158 treats Human African trypanosomiasis (HAT), caused by *Trypanosoma brucei*. From in vivo testing, SCYX-7158 was found to be safe, efficacious, and orally active. DNDI-6148 attacks Visceral leishmaniasis (VL), a severe infectious disease caused by protozoan parasites *Leishmania donovani* and *Leishmania infantum.* DNDI-6148 is potent and active in vivo and in vitro by targeting *Leishmania* CPSF3. Figures are created using ChemDraw.

Benzoxaborole was first synthesized by Anacor Pharmaceuticals [134]. Compared to B-aryl boronic ester compounds, benzoxaborole displayed much higher polarity, excellent antifungal properties [26,125], and could host a variety of aryl substitutions. Since then, the benzoxaborole structure has been used as a template for other drugs to combat parasitic infections. Notable examples include AN3661, SCYX-7158 (AN4169) and DNDI-6148 (Figure 35), which treat malaria [135], Human African trypanosomiasis (HAT) [79], and Visceral leishmaniasis (VL) [95], respectively.

**Figure 36:**
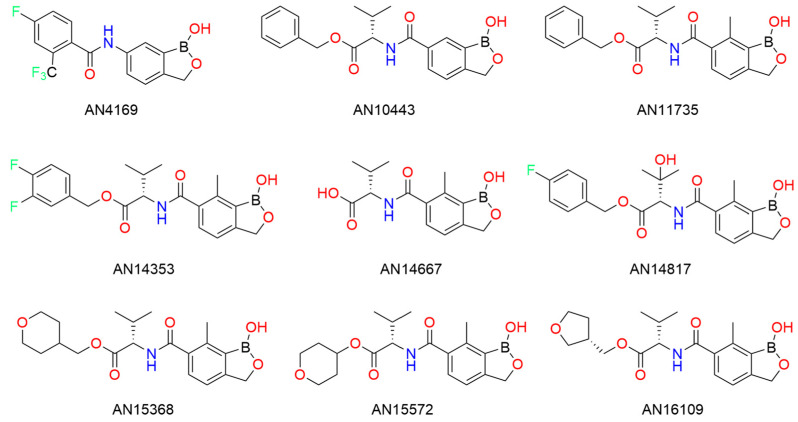
Chemical structures of all benzoxaborole candidates. In this study, the progression of candidates was AN4169 to AN10443 to AN11735 to the final candidate, AN15368. While AN4169 cured all mice infected with *Trypanosoma cruzi* Brazil strain, the low therapeutic margin encouraged investigation of alternative benzoxaboroles. Development from AN10443 to AN11735 was motivated by results from in vitro testing on a mouse liver fraction. After in vivo tests, AN15368 was selected as the most promising candidate for clinical trials. Figures are created using ChemDraw.

From in vitro testing and structure-activity relationships (SARs), nine benzoxaborole analogues were produced and compared in terms of effectiveness (Figure 36). Padilla et al. [133] first analyzed benzoxaborole AN4169. While AN4169 cured all mice infected with *T. cruzi* Brazil strain, its narrow therapeutic window could threaten host health. Simultaneous studies evaluating AN4169 analogues on *Trypanosoma congolese* provided stable benzoxaborole derivatives that could target *T. cruzi* in a mouse S9 liver fraction assay, such as AN10443. Compared to AN10443, AN11735 with a methyl group at C(7) demonstrated far superior in vitro activity, while larger substituents reduced efficacy. Ester functionality was crucial for observing drug activity. Interestingly, esters with basic amines were less effective than their neutral counterparts, while most aliphatic esters had high success. Thus, a general trend was found: the hydrophilic compounds with water solubility were found to be less metabolized in the S9 liver fraction.

Beyond high in vitro activity, candidates with valine esters (such as AN15368 and AN16109) were stable in mouse and human S9 liver fractions. They also demonstrated advantageous pharmacokinetic properties, such as low hepatic clearance from blood and bioavailability at low dosages, regardless of intravenous or oral administration. AN14353 was a standout candidate demonstrating high potency, rapid in vivo activity in infected wild-type and immunodeficient mice.

The importance of the ester group was further highlighted by the inactivity of amide, N-methyl amide, ketone, ether and acylsulfonamide analogues of AN11735. Interestingly, carboxyllic acid analogue AN14667 had 1000-fold reduced intra- and extracellular activity. Padilla et al. suspected that ester-containing benzoxaboroles were prodrugs, cleaved by a host or parasitic enzyme into their carboxylic acid counterpart. Ester-cleaving enzymes in *T. cruzi*, including serine carboxypeptidase, were identified and ablated by CRISPR/Cas9. Wild-type *T. cruzi* converted AN15368, while knocked *T. cruzi* could not, affirming that prodrug conversion is driven by serine carboxypeptidase-dependent prodrug in *T. cruzi*.

To optimize metabolic stability, analogues of AN14353 with more polar and non-basic substituents were tested. Compounds with the best in vitro activity were further evaluated for pharmacokinetics and metabolic stability through in vivo tests and in vitro experiments using the S9 liver fraction and plasma of humans and mice. The results led to the discovery of three more promising compounds: AN14817, AN15368, and AN16109. All three compounds were found to be potent and safe, with negligible affinity for a variety of mammalian enzymes, receptors, and ion channels. They were also non-genotoxic and did not inhibit cytochrome P450 enzymes at a concentration of 10 μM. However, in 7-day toxicology studies in rats, AN15368, with higher hydrophilicity, had superior dose-proportional exposure in rats, limited hematological effects (below 120 mg kg^−1^d^−1^), and no evidence of drug accumulation.

AN15368 was selected as the best candidate for testing in naturally infected *Macca mulatta* non-human primates. Macaques were infected and observed during a 60-day treatment period, similar to the time period for human treatments and clinical trials [136,137]. Efficacy was examined using three criteria: presence of parasite DNA in blood, presence of parasite DNA in post-necropsy tissue samples and antibody levels for *T. cruzi* recombinant proteins. After treatment, macaques were observed in 2-to-4.5-week intervals. AN15368-treated macaques tested negative for parasitic DNA or culture, while two of three untreated animals were positive at multiple intervals. Similarly, parasite DNA was absent in post-necropsy tissues of nine treated macaques. Nine of the ten remaining treated macaques were monitored periodically for over 3 years. All treated animals demonstrated lower levels of anti-*T. cruzi* antibodies, while blood PCR and hemoculture consistently tested negative for *T. cruzi* presence. During the treatment period, the macaques readily took treats containing AN15368 and showed no drug-related symptoms or side effects. Moreover, female macaques appeared fit and produced healthy infants.

During the study, successful analogues of AN15368 treated African trypanosomiasis by targeting the Cleavage and Polyadenylation Specificity Factor (CPSF3), an essential factor in mRNA processing [97,138]. It was also found that overexpression of CPSF3 increases AN15368 resistance by 3 to 5 times. In this study, parasitic mRNA levels of AN14353-treated cells reduced following 6 h of exposure, while benznidazole-treated cultures displayed consistent parasitic mRNA levels. The high selectivity and precision of AN15368 can be attributed to specific activation by parasitic carboxypeptidase enzymes, resulting in a cleaved product that targets CPSF3 in *T. cruzi* and interferes with parasitic mRNA processing.

AN15368 has proven to be a reliable and highly effective treatment with little to no negative side effects, and it is the first treatment for Chagas disease that is both highly effective and orally bioavailable. Since T. cruzi infects a wide range of animals in similar ways, results seen in macaques may apply to humans. Benzoxaboroles offer a great starting point for developing new treatments against protozoal infections by targeting the mRNA processing endonuclease CPSF3 [139,140]. Future drugs can build on the benzoxaborole framework to target other protozoal infections.

## 15. Boron Encapsulated in a Liposome for Neutron Capture Therapy

BNCT is a targeted type of radiotherapy that effectively treats tumors in cases of inoperable or recurring malignant tumors, including those in the brain, head, neck, and skin. Typically, Boron-10 is concentrated in tumour cells and then exposed to irradiation from a neutron beam [141]. A nuclear reaction occurs, producing alpha particles (Helium-4) and lithium-7 nuclei, which damage the DNA within tumour cells, thereby eliminating them [141,142]. However, current boron delivery agents often exhibit low in vivo stability, low biocompatibility, and limited combinational modalities, all of which reduce their clinical application [143]. The boronated tyrosine derivative 4-boronophenylalanine (BPA), the most common boron delivery agent, results in inadequate boron uptake by the tumour. Therefore, there is a need for highly tumor-specific boron delivery agents to improve BNCT treatments. To address the current limitations of existing BNCT, Li et al. [143], introduced Boronsome as a modified liposome with drug delivery capabilities. Boronsome carrying Olaparib has proven to be a non-toxic and effective anticancer treatment for BNCT treatments.

Boronsome is a liposome made from carboranyl-phosphatidylcholine, which is derived from carborane. Carborane is an icosahedral cluster consisting of two carbon atoms, twelve hydrogen atoms, and ten boron atoms [144]. It is well-known for its thermal and redox stability, high hydrophobicity, and low nucleophilicity. In comparison to previous liposome structures, Boronsome contains a greater number of boron molecules, thereby enhancing the boron concentration effectively in the targeted tumour cells [143].

Boronsome serves as an effective delivery agent in positron-emission tomography (PET) imaging-guided BNCT while concurrently applying chemoradiotherapy. Theoretical simulations and experimental tests indicate that boronsome exhibits high stability and significant tumour accumulation. It remains stable in 50% bovine serum for over three days, and cancer cells treated with boronsome absorb 61% more boron compared to BPA. Overall, boronsome inhibits tumour growth during BNCT and shows enhanced efficacy when combined with chemotherapy drugs.

**Figure 37:**
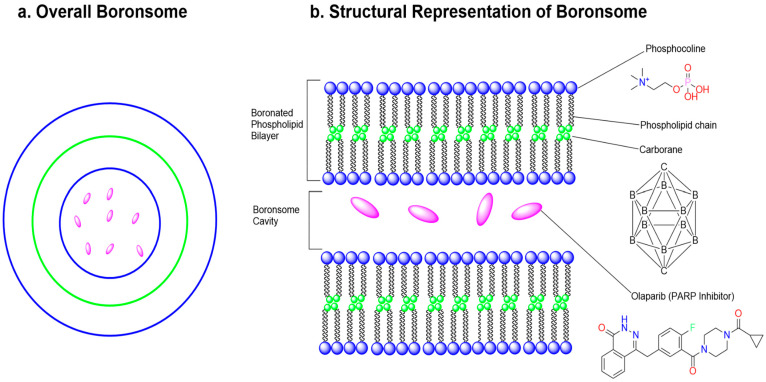
Two-dimensional representations of overall Boronsome structure (**a**) and internal Boronsome membrane (**b**) containing boronated phospholipid bilayers and internal cavity with olaparib PARP inhibitor. The boronated phospholipid bilayer used is BoP-3, consisting of phosphocholine, phospholipid chain, and carborane. Boronsome can be a vehicle for other chemotherapy drugs. Figures are created using ChemDraw.

Additionally, Li et al. [143] aim to produce boronosomes by covalently bonding carboranyl, derived from carborane, to the hydrophilic tail of phospholipids, resulting in boronated phospholipids (BoPs). The hydrophilic head phosphocholine was retained to enhance biocompatibility and minimize toxicity [145]. A key feature of boronosomes is the placement of boron within the liposome structure, creating space in the cavity for other drugs (Figure 37). Carborane features a three-dimensional icosahedral structure, while the hydrophobic tails of phospholipids usually form a two-dimensional configuration. Due to potential steric hindrance between the bilayers, four boronated phospholipids of varying lengths were synthesized. Molecular dynamics (MD) simulations revealed that BoP-3, the smallest bilayer, exhibited the best stability and cargo-carrying capacity, attributed to its increased thickness, enhanced intramolecular hydrogen bonding, and greater order in the palmitoyl side chains. Additionally, BoP-3 showed the highest cargo loading efficiency in experimental tests using Sulforhodamine B (SRB) encapsulation. In addition, the transmission electron microscopy indicated that the diameter of the boronsomes is 100 nm, with a bilayer thickness of 15.78 nm.

Boronsome was tested using in vitro methods to assess cellular boron concentration in triple-negative mouse breast cancer 4T1 cells. This evaluation aimed to determine the effectiveness of boron concentration in tumour cells. Observations indicated that boron concentration increased with boronsome dosage, with the highest dosage (5 mg/mL) yielding a boron concentration of 182.5 ppm, which exceeds the minimum threshold for clinical BNCT to ensure tumour death: $1\times 10^{9}$ boron-10 atoms per cell or 20 μg/g of tissue [146]. PET imaging was employed to analyze boron concentration throughout the mice. Moreover, boronsomes were labeled with radioactive nuclides and incorporated radiolabeled lipids in the bilayer to monitor boron concentration. Boronsome demonstrated stability for up to 24 h and did not affect cell viability at any concentration.

Following treatment, female mice with 4T1 tumors received radiolabeled boronsomes for 6–8 weeks. BNCT was performed on the upper regions of the mice. The boronsomes concentrated in the tumour, resulting in a favourable Tumour/Normal (T/N) ratio, while their accumulation in the lungs, muscles, brain, fat, bones, heart, and blood remained relatively minimal. Over 21 days, the treatment, which combined boronsomes with neutron irradiation, led to tumour reductions, achieving complete eradication in 4 out of 9 mice. No toxicity was observed from this treatment. These findings underscore the efficacy and biospecific nature of boronsome-BNCT.

Additionally, high-LET particles in BNCT are known to damage DNA [147] and its repair mechanisms. Poly (ADP-ribose) polymerase-1 (PARP1) is a key enzyme in DNA strand repair and is recognized for enhancing the effectiveness of radiotherapy [148]. For this reason, Li and his group incorporated olaparib. The FDA-approved PARP inhibitor, olaparib, was encapsulated in boronsomes and combined with BNCT to disrupt the DNA repair processes of cancer cells. A colony formation assay revealed that cell viability with PARP inhibitors was nine times lower than that of samples treated with boronsomes and BNCT alone. PARP inhibitors enhance efficacy by interfering with DNA repair, leading to increased cell death. Consequently, Li et al. [143], successfully manipulated liposomes with carboranes to form a novel carrier for the anticancer drug. The resulting molecule, boronsome, has high specificity for tumour cells and is highly effective in halting tumour cell growth. The effectiveness of boronsome is amplified when used in BNCT treatment while carrying PARP inhibitors.

Li et al. have developed boronsome, a modified liposome containing carborane and phosphocoline, which overcomes many limitations of current boron delivery agents that suffer from poor stability, compatibility, and limited options for combination therapies [143]. Boronsome is a new treatment and delivery system capable of selectively transporting enough boron-10 to tumour cells. Tumour growth can be further reduced when boronsomes carry PARP inhibitors like Olaparib. Overall, boronsomes offer the potential to enhance BNCT treatments by lowering costs, improving results, and expanding the possibilities for combined therapies.

## 16. Boron Nitride for Combating Resistant Bacteria

Antimicrobial resistance is a growing global health threat that affects both humans and animals [149]. Due to the emergence of resistant microorganisms, common infectious diseases are becoming increasingly challenging to treat, healthcare costs are rising, illnesses persist, and mortality rates worldwide are increasing. Microbes, including bacteria, fungi, parasites, and viruses, develop resistance mechanisms against antimicrobial drugs through evolutionary adaptations, such as enzyme modification and biofilm formation [150]. Current antimicrobial agents include polysaccharides, peptides, glycopeptide polymers, and engineered nanomaterials (ENMs). While some nano-antibacterial agents have been tested, many have proven to have poor biosafety, failing to distinguish between target microbes and mammalian cells. Pan et al. [149], developed two-dimensional boron nitride (BN) nanosheets as an effective and versatile nano-antimicrobial treatment, even towards antimicrobial-resistant (AMR) bacteria. In mammals, BN nanosheets demonstrated excellent in vivo safety and failed to trigger secondary resistance after long-term dosage. BN nanosheets selectively target bacterial surface proteins, such as FtsP, EnvC, and TolB, thereby preventing Z-ring constriction and ultimately inhibiting cell division.

Engineered nanomaterials (ENMs) have at least one dimension smaller than 100 nanometers and are manufactured at a near-atomic scale using nanotechnology [151]. Two-dimensional boron nitride (BN) nanosheets have been found to be an effective antimicrobial agent. Initially, the physical properties of the ENMs were examined using transmission electron microscopy (TEM), atomic force microscopy (AFM), Raman spectrometry, dynamic light scattering, and electrophoretic light scattering.

Following this, the antibacterial properties of all ENMs were assessed. The cell environment consisted of E. coli with ampicillin resistance and norfloxacin vulnerability. The growth patterns of the resistant E. coli were monitored after 24 h for all ENMs, with doses ranging from 0 to 500 micrograms/mL. The ENMs in the leftmost column of Table 2 exhibited promising dose-dependent antibacterial effects, whereas the others showed limited efficacy. As shown in Table 2, BN nanosheets and silver nanoparticles (Ag NPs) had the lowest minimum inhibitory concentrations (MICs).

One of the main challenges for nano-antimicrobial agents is their toxicity to mammalian cells. To better understand the safety of Ag NPs and BN nanosheets in different mammalian cells, Pan et al. [149], used colorimetric quantification via MTS cell proliferation assays. The researchers tested the biosafety of these agents across three types of mammalian cells: macrophage-like cells (THP-1), epithelial cells (HUVEC), and mouse bone marrow-derived dendritic cells (BMDCs). The results showed that BN nanosheets were much safer, with cell viability over 80% in all three types of cells. Notably, the cells were able to adapt to doses as high as 500 µg/mL. In contrast, Ag NPs had poorer biosafety, with less than 20% cell viability in THP-1 and BMDCs. Furthermore, they investigated the biosafety of BN nanosheets in vivo through intravenous injection and oropharyngeal instillation. They also examined the acute inflammatory effects of BN nanosheets and Ag NPs in lung, heart, liver, kidney, and spleen tissue after 24 h using Hematoxylin and Eosin (H&E) staining. The results indicated that BN nanosheets were not significantly toxic to mammals and did not trigger major acute inflammation in the tested organs, suggesting a precise mechanism targeting bacteria. However, Ag triggered severe pulmonary inflammation.

Researchers took several steps to identify the specific mechanisms and molecular targets of BN nanosheets. They used TEM to study the distribution of these materials within cells. The results showed that both Ag NPs and BN nanosheets were bound to the cell membranes but remained outside the cell. BN nanosheets did not enter the cytoplasm, although they were found to puncture the cell wall and interact with proteins in the periplasm. With super-resolution microscopy imaging (SRM) and HADA fluorescence, the team found that BN nanosheets disrupted the peptidoglycan layer of the bacterial cell wall. Unlike Ag, BN nanosheets seemed to stop binary fission before the cells split into daughter cells. This was confirmed by scanning electron microscopy, which revealed that BN-treated cells had sausage-shaped envelopes, indicating cell cycle obstruction.

**Figure 38:**
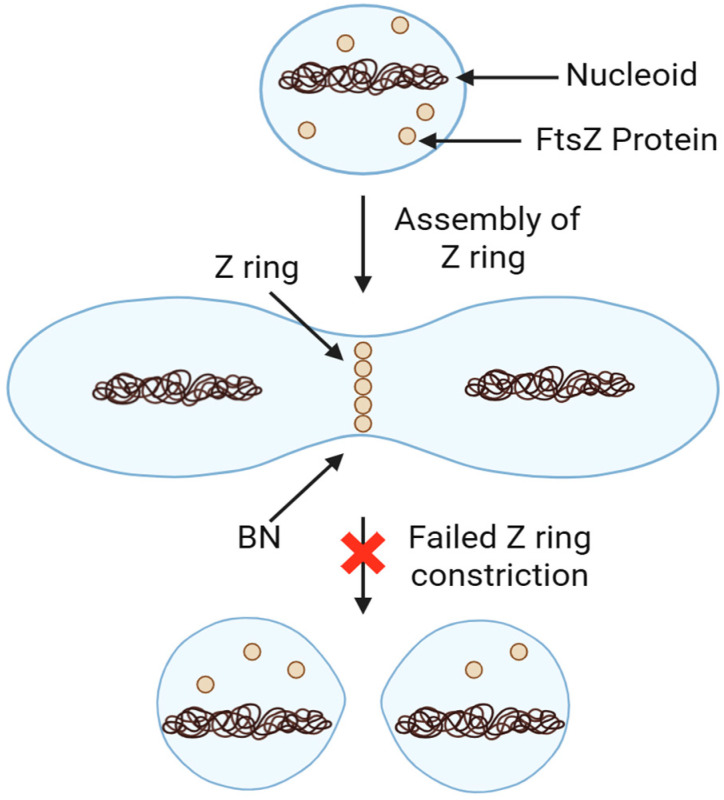
Mechanism of BN nanosheets in bacterial cell division. In the latter stages of bacterial cell division, FtsZ proteins assemble into a ring, known as the Z ring. Typically, the Z ring serves as a scaffold to attract cell division proteins and generates a contracting force to facilitate cytokinesis. However, BN nanosheets interfere with Z-ring constriction, disturbing the final steps of bacterial binary fission. Figures are created using BioRender, Created in BioRender. Deb, M. (2025) https://BioRender.com/mut1fd3.

Notably, *E. coli* cell duplication begins with the formation of FtsZ, a bacterial protein similar to tubulin that creates the Z ring [152]. Usually, the Z ring behaves as a scaffold to invite cell division proteins and provides a contracting force to provoke cytokinesis [153]. Later, FtsZ is rapidly broken down in *E. coli* by ClpXP protease [154]. Pan et al. proposed that BN nanosheets interact with cell division proteins thereby disrupting the cell cycle [149]. To investigate this, Pan et al. examined how BN affects Z-ring activity using fluorescent anti-FtsZ labelled with fluorescent Alexa Fluor 488. Untreated cells showed low FtsZ levels, whereas cells treated with BN had a significant accumulation of FtsZ. Additionally, about 13.97% of cells treated with BN had Z rings, compared to 3.59% in the control, confirming that BN nanosheets interfere with the constriction of Z rings during cell division (Figure 38).

Since Z ring constriction is controlled by many membrane proteins, the authors developed “biotin labelling of the surface proteome” (BLSP) to decipher the specific BN-targeted proteins. Similar tests were performed with ampicillin, Ag, and cellulose nanofibers (CNFs). BN-targeted proteins would be expected to be low in abundance; BN-treated cells, 95 of 303 proteins were down-regulated, with 15 of them directly associated with cell division. Meanwhile, 14 cytoplasmic proteins with involvement in respiration, metabolism, and biosynthesis were up-regulated, possibly to signal cell cycle arrest.

Finally, in vivo testing was performed in mouse lung cells infected with levofloxacin-resistant *P. aeruginosa* bacteria. From observing luminescence signals, mice treated with PBS (vehicle control) and levofloxacin still demonstrated a significant presence of bacteria within the lungs. Levofloxacin could reduce blood leakage within the lungs, but worsen inflammation. BN nanosheets, however, had one-third of the colony-forming units compared to PBS and levofloxacin treatments. The lungs also had less blood leakage and inflammation. In terms of overall survival, mice treated with BN nanosheets had a 50% chance of survival, 25% better than just levofloxacin and PBS. Mammalian cells can endocytose BN nanosheets into lysosomes, so almost all BN nanosheets were naturally removed from the survivors’ lungs after 7 days. While BN nanosheets are less cytotoxic to mammals than silver nanoparticles, BN nanosheets delivered by intravenous injection can result in liver damage.

Pan and his group comprehensively tested the antibacterial efficacy of BN nanosheets. In preliminary testing, BN nanosheets demonstrated high antibacterial efficiency against five resistant bacterial strains and retained their impact after multiple generations, thereby preventing the development of evolutionary microbial resistance. Among the 16 tested nanoparticles, BN nanosheets were the only treatment to selectively target Z-ring constriction in bacterial cells, resulting in the specific interruption of bacterial duplication. Finally, in vivo testing in resistant *P. aeruginosa* animal lung cells also yielded positive results. Compared to other antibiotics, cells treated with BN nanosheets were twice as likely to survive. Overall, BN nanosheets are a unique nanoantibacterial agent with high bacterial selectivity, evolutionary resistance, and high efficacy. Given that other nanosheets like graphene oxide and graphene behave differently depending on orientation, investigation of the orientation and ionization of BN nanosheets may lead to interaction with more membrane components.

## 17. Post-Translational Insertion of Boron in Proteins

Despite B being an essential micronutrient [155], B is not found in amino acids or proteins [156]. Chemically, B shares similarities to carbon (C) and silicon (Si) and has unique electron-pair-accepting capabilities rarely found in biological systems [157]. Mollner et al. [156], discovered boron reactivity in proteins by post-translationally introducing Cβ–Bγ bonds in boronyl amino acid boronoalanine (Bal), thereby providing opportunities for site-selective, dative Lewis acid-base pairing (LABP) within protein complexes. Configurable protein-LABP permits adjustable intermolecular and intramolecular ligand-host interactions, while reactive protein-LABP reveals reactive sites formed from Cβ–Oγ covalent bonds. Ultimately, these dative-bonding interactions revolutionize protein function by enabling control of thermal and proteolytic stability, observation of structural features by chemical exchange, and artificial post-translational “substitution mutation” of Bal to Serine (Ser) [156].

While B shares similar electronic properties to C, it is not as prevalent in biological systems as typical organic life elements such as carbon (C), oxygen (O), nitrogen (N), and hydrogen (H). Many of B’s unique properties stem from its selective, dative interactions with ligands, typically O or N-containing Lewis bases, which can even be observed in competing Lewis-basic solvents like water (H_2_O). B can interchange between two semistable binding states: electron-deficient ligated Lewis acidic sp2 (6e-) or electron-rich dative bonded sp3 (8e-) [156]. Also, nearby functional groups can alter the Lewis acidity of B. For example, Wulff-type boronates are characterized by an intramolecular dative bond between B and N, reducing the pKa of B [158].

**Figure 39:**
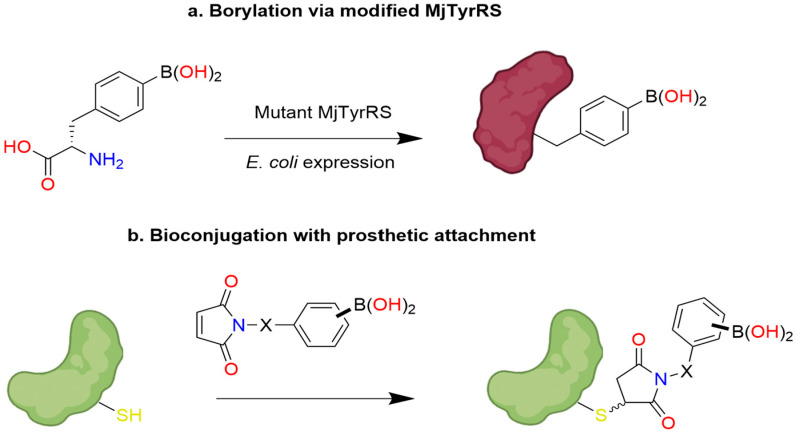
Cartoon illustration of published borylation methods. Green and red clusters represent example proteins. (**a**) Addition of *p*-boronophenylanalanine to introduce boronic acid in *E. coli* proteins. *Methanococcus jannaschii* tyrosyl-tRNA synthetase (MjTyrRS) is altered to associate tRNA_CUA_ with an unnatural amino acid. (**b**) Bioorthogonal boronate formation on proteins through conjugatable 2-methyl-5-carboxymethylphenylboronic acid. The prosthetic group can interact with the thiol in cysteine in a click reaction. Figures are created using BioRender and ChemDraw. Created in BioRender. Deb, M. (2025) https://BioRender.com/mut1fd3.

Currently, the process of natural borylation cannot be predicted, controlled, or manipulated due to its dynamic behavior in biomolecular systems. B insertion methods in biomolecular systems are limited and can be sequestered by Lewis bases in biomolecules. Two recently published methods (Figure 39) for site-specific borylation are incorporation via modified *Methanococcus jannaschii* synthetase (MjTyrRS) [159] or bioconjugation with prosthetic attachments [160,161]. However, both methods have limitations, which include reduced protein translation yields and the requirement of bulky linker molecules, respectively [156].

**Figure 40:**
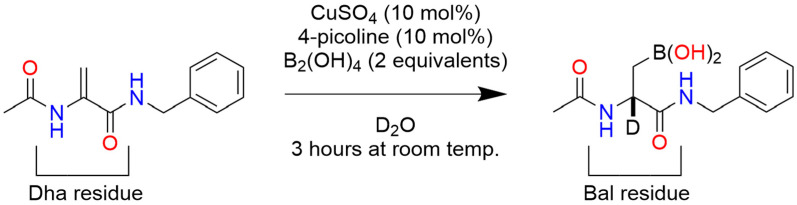
Optimal reaction conditions for creating boronoalanine (Bal) in model peptide 2-acetylamino-*N*-benzyl-acrylamide containing dehydroalanine (Dha) residue. Beyond high yield (Table 3), high regioselectivity of 98% for Cβ–Bγ bond formation further highlights the excellent precision of this scheme. Figures are created using ChemDraw.

Unlike nonanchored B-O bonds, Mollner et al. hypothesized that B-C bonds can be predictable and stable enough to support the precise insertion of B(OH)_2_ [156]. This is the case for boronoalanine (Bal) residue, which contains an alkylborono group, but is challenging to isolate. Therefore, they further explored Bal formation via selective hydroborylation of the terminal alkene found in dehydroalanine (Dha), a versatile, biocompatible amino acid residue [162]. Firstly, the reactivity of Dha within the small model peptide in 2-acetylamino-*N*-benzyl-acrylamide was assessed. To optimize borylation, various ligands, copper sources, and bases were tested to optimize Cu-mediated borylation of Dha with tetrahydroxyboron B_2_(OH)_4_. Most copper (II) sources (such as CuSO_4_ and Cu(NO_3_)_2_ with 4-picoline additive gave exceptional yields (Table 3). The most optimal reaction scheme, with CuSO_4_ and 4-picoline, is shown in (Figure 40).

**Figure 41:**
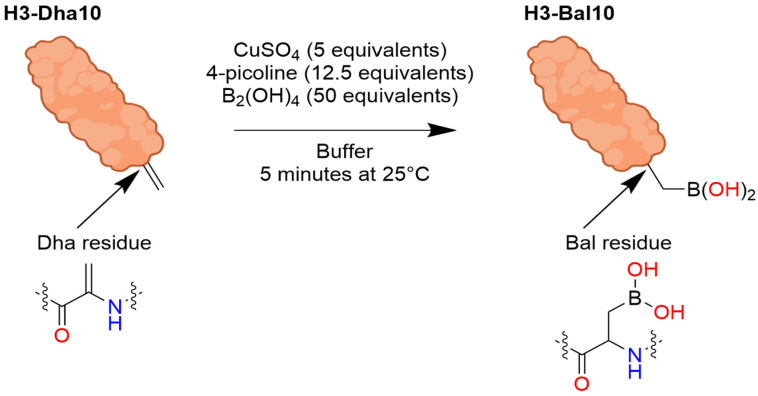
Cartoon illustration of optimized reaction conditions for borylation of Histone H3 at 10th position from dehydroalanine (H3-Dha10) to boronoalanine (H3-Bal10). Compared to optimal conditions for model peptide 2-acetylamino-*N*-benzyl-acrylamide (Figure 40), borylation of H3-Dha10 uses more CuSO_4_ (50x), 4-picoline (125x) and B_2_(OH)_4_ (25x), for 5 min at 25 °C. Instead of deuterium water (D_2_O), a buffer solution (pH of 7.0) containing 100 mM of Sodium Phosphate buffer (NaP_i_) and 3 M of guanidine hydrochloride were employed. Figures are created using BioRender and ChemDraw. Created in BioRender. Deb, M. (2025) https://BioRender.com/1l1ln0y.

Afterward, the borylation scheme was optimized in protein context, specifically the H3 protein histone. The most optimal conditions (Figure 41) yielded excellent conversions to H3-Bal10 >90%. Histone H3-Dha10 was most reactive at pH 6.5–8.5, further enhanced by common denaturants guanidine hydrochloride or urea. A buffer solution (pH of 7.0) with 100 mM of Sodium Phosphate buffer (NaP_i_) and 3 M of guanidine hydrochloride was selected. Some reactants were scaled up: 50 equivalents of B_2_(OH)_4_, 5 equivalents of CuSO_4,_ and 12.5 equivalents of 4-picoline.

Next, borylation was tested on a variety of proteins with different sizes, folds, stability, and biological functions (Table 4). In all cases, Bal was successfully and selectively inserted into the proteins, without hindering original folds, properties, and functions.

Upon establishing reliable and selective Bal insertion methods, subsequent experiments assessed the behaviour of Bal within proteins and complex biomolecular environments. Depending on kinetics and external modulation, alkyl boronates behave as Lewis acids near Lewis bases to form LABP. Interestingly, Bal has higher acidity (pKa = 8.31) relative to alkylboronic acids (e.g., methylboronic acid, pKa = 10.7) and alkylboronic acids (e.g., phenylboronic acid, pKa = 8.8). Using protein mass spectra, Bal occurred with different levels of boronyl substitution (free, mono, and di) in each unique protein and site (Table 4), due to differences in Lewis base environments. Further investigation using protein NMR confirmed that boron in Bal could form pH-dependent reversible interactions with peptide backbone carbonyl oxygens, forming oxaboralane.

Intramolecular interaction with backbone carbonyls suggests Bal can engage with intermolecular Lewis bases and Wulff-type interactions. Proteins with natural binding abilities, PstS and Annexin V, were compatible with Bal while maintaining their original binding function. On the contrary, histone H3, which cannot behave as a host, had Bal inserted at position 10 to observe new intermolecular interactions with biologically derived poly-ols (terpenes and glycans). From 19F NMR, H3-Bal10 engaged in intermolecular PLABP, where ligands competed reversibly and selectively for the host site. Site selection bias was also observed, where H3-Bal9 was a better host to diols than H3-Bal10. New binding abilities with Bal could be exploited to detect diol. Glycolysated mammalian cells (CHO) were tested with original and Bal-modified red fluorescent protein mCherry. Flow cytometry analysis revealed that nonborylated mCherry had minimal binding (<5%) while borylated mCherry-Bal131 had significant binding (>80%), demonstrating cell-surface recognition by direct binding with great relevance to carbohydrate/sugar sensing.

Successful utilization of Bal in PLABP can also be used to “flag” protein interactions. Many reactive oxygen species (ROS) can behave as alpha-nucleophiles, capable of converting Bal to Ser courtesy of PLABP and subsequent C-O bond formation. This interaction acts as a “flag” to signify interaction but also allows for synthesis of Dha to Ser via Bal, enabling post-translational change and formation of βC–γO. To test this, H_2_O_2_ was used as a representative ROS. As shown in Table 5, reactive sites of some Bal-modified proteins demonstrated accessibility to H_2_O_2_, with all but Npβ-Bal61 and PstS-Bal197 being relatively accessible to ROS. Furthermore, MS/proteolytic-MS confirmed conversion of Dha to Bal to α-deuterated Ser, allowing for “post-translational mutations”. The deuterium label can also help with spectroscopic and mechanistic analyses.

After demonstrating PLABP in single proteins, the next step was to assess interactions in more complex systems. Since natural borylation tends to provide stability, the thermo and proteolytic stability of Bal proteins could be altered, potentially chemoselectively. Three proteins were modified with different levels of intramolecular engagement: partially engaged PsTS-Bal197, strongly engaged Annexin V-Bal316 and multiply engaged Npβ-Bal61. Overall structure and function for each protein remained the same, but thermal tolerance could be changed. With differential scanning fluorimetry, the melting range, the difference between onset temperature (*T_ON_*) and melting temperature (*T_M_*) could be assessed. PstS-Bal197 and Annexin V-Bal316 had larger differences in their melting transitions (*T_ON_*-*T_M_*) of +16 °C and +21 °C, respectively, while multiply engaged Npβ-Bal61 had a lower melting range of −6 °C.

The proteolytic stability control of PLABP was assessed in the multi-protein SUMOylation cascade. Small ubiquitin-like modifier (SUMO1) regulates nuclear transport and cell cycle progression [163]. Normal SUMOylation is controlled by the protease SENP1, where zymogen pre-SUMO1 is converted to SUMO1, which is subsequently converted to matured SUMO1. Bal was inserted to make pre-SUMO1-Bal51 to assess stability in SUMOylation. While pre-SUMO1 is susceptible to degradation by serine protease chymotrypsin, pre-SUMO1-Bal51 proved much more stable, while being compatible with cysteine proteases and ligases needed for the full SUMOylation cascade. Matured SUMO-1 could still be produced despite the introduction of stabilizing Bal. Ultimately, Bal insertion with PLABP improved proteolytic stability without hampering the conventional cascade sequence and functionality.

Currently, Paramagnetic relaxation enhancement (PRE) is flawed due to a lack of short-range quantitative information and the requirement of a spin label, which can interfere with structure. From prior tests, Bal has a propensity to interact with carbonyls transiently and reversibly, forming dative contacts. By controlling signal intensity by PLABP chemical exchange, this interaction may bestow details on structure and interaction through protein NMR. Dative-contact induced chemical exchange (DICE) could be a valuable, benign method to measure protein contact. To observe this, Bal was treated as an NMR probe in proteins with higher-order structures. Upon spectral observation of denatured H3-Bal9 and H3-Ser9, most residues had similar intensities, but in 13CO-15N correlation spectra, six peaks in H3-Bal9 were completely absent due to a transient carbonyl-boron interaction that reduces signal intensity and is amplified with higher temperatures. Ultimately, signal loss can help describe the extent and location of between Bal and the backbone without disrupting the overall structure and other spectral signals.

Afterwards, DICE was tested against PRE to observe transient interactions, which are relevant to disordered, multi-state proteins. While PRE usually can only detect long-range contacts, DICE shows region-specific signal intensity loss and showcases transient long-distance contacts and short-range contacts. DICE could also be used on a more complex system, like a nucleosome, consisting of a histone octamer of two H2A, H2B, H3 and H4 proteins each and wrapped by DNA. While central sections of the nucleosome can be observed in high resolution, histone tails are difficult to discern because of their disordered nature and potential post-translational modifications. A nucleosome was created with 15N-labelled, borylated H3 to create 15N-H3-Bal9. HP1 is recruited by trimethylation of Lys9, while Ser-10 phosphorylation is performed by Aurora B kinase and leads to HP1 ejection. Ser-10-phosphorylation-dependent HP1 recruitment at Lys9, so this position of Bal was strategic to observe phosphorylation-driven structural change. With 1H-15N-HSQC spectra, precise structural information could be collected on the intact nucleosome. Here, the spectra revealed a “double dip” pattern, indicative of two α-helices (H3 positions 3–10 and 17–28) connected by a β turn. While this observation wouldn’t be possible with the wild-type, Bal shows specific short-range interactions, which can help identify nearby structural changes. Given that Bal did not impact structure, the structural changes of phosphorylation could be observed. In the histone H3 tail, temporary folds were lost, histone tail flexibility increased, and intramolecular PLABP interactions decreased, suggesting that phosphorylation “decompressed” the tail.

Often in nature, borates (O-B-R) are much more prevalent than boronates (C-B-R), but neither are found in proteins. In conclusion, Mollner and his group investigated the binding and functionality of C-B containing Bal residue. Efficient methods for optimized Bal insertion into peptides and proteins have proven more effective than past borylation techniques. In proteins, Bal was involved with PLABP, allowing for unique intermolecular and intramolecular reactions with fascinating applications, including diol sensing, post-translational Ser insertion, adjustable proteolytic and thermal stability, and structural analysis by DICE. Interestingly, Bal exists as a mixture of L/D stereoisomers in unpredictable ratios, ranging from 2:1 to 1:1. Future studies can assess the functionality and interactions of both isomers and employ ligands and catalysts to provide stereospecific control. While this study primarily examined binding with naturally sourced ligands, additional practicality may be observed through experimentation with synthetic ligands.

## 18. Boron-Based Probes Used in Biological Imaging

**Figure 42:**
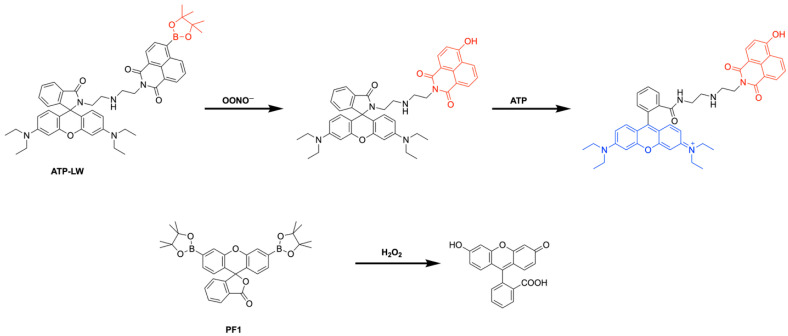
Sensing mechanism of the cell-permeable fluorescent probes ATP-LW and PF1 as they react with peroxynitrite (OONO^−^), ATP, and hydrogen peroxide (H_2_O_2_) chemoselectively. The dual-analyte function of ATP-LW allows for simultaneous monitoring of ATP and OONO^−^ in vivo. PF1 reacts chemoselectively with hydrogen peroxide. Figures are created using ChemDraw.

**Figure 43:**
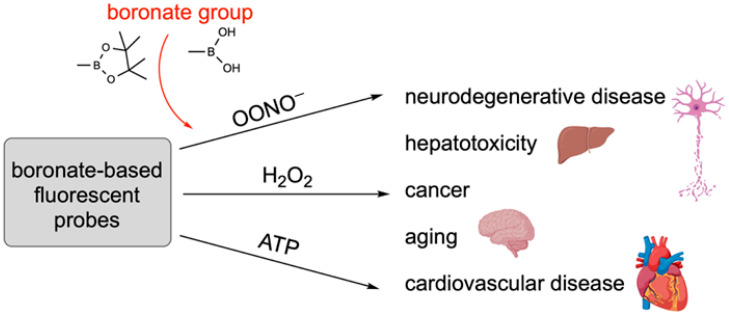
Boron-based fluorescent probes can react selectively with peroxynitrite, hydrogen peroxide (H_2_O_2_), and ATP depending on their design. This enables in vivo monitoring of oxidative stress, which has been implicated in various disease mechanisms, including neurodegenerative diseases and aging. Figures are created using ChemDraw.

Recent advancements in fluorescent probes for the bioimaging of reactive nitrogen species (RNS) and ROS have also made use of boron-containing organic compounds such as boronic acids and esters. In 2005, Miller and team developed the Peroxysensor family, a class of fluorescent probes capable of permeating the cell and responding chemoselectively to H_2_O_2_ on the micromolar scale (Figure 42) [164]. Additionally, several fluorescent probes have been developed that rely on boron’s specific susceptibility to nucleophilic attack by peroxynitrite (ONOO^−^), allowing for the monitoring of peroxynitrite stress in vivo. The sensing mechanism of these peroxynitrite probes relies on the cleavage of the boronic ester by peroxynitrite, leaving either a hydroxyl group or cyclizing to form fluorescent reaction products [165]. In 2021, Luling et al. developed ATP-LW, a fluorescent probe capable of detecting both ATP and OONO^−^ simultaneously, which they used to examine the fluctuation of ONOO^−^ and ATP in hepatotoxicity (Figure 42) [166]. The use of boronate-based probes for hydrogen peroxide or peroxynitrite allows for the observation of oxidative stress in vivo, which has been associated with neurodegenerative disorders such as ALS, along with cancer and aging (Figure 43) [167,168].

## 19. Toxicity of Boron-Containing Compounds

Boron is an essential micronutrient in plants, involved in cell wall integrity and various metabolic functions [169]. It has also been associated with bone development, hormone metabolism, and immune function in humans and other animals [170,171,172]. The World Health Organization (WHO) estimates a safe daily intake range of 1.0–13 mg/day in adults [173]. Boric acid, a metabolic byproduct of boron-containing compounds, exhibits an oral LD50 of 3450 mg/kg and 4080 mg/kg in male and female rats, respectively, comparable to that of table salt [174,175]. However, excessive boron intake is known to cause toxicity in both preclinical models and in humans. Boric acid has demonstrated both inhalation and dermal toxicity in those subjected to excessive exposure in the workplace or treated with large quantities of boric acid ointment/solution over a prolonged period of time. Utilizing reports on accidental poisonings by boric acid, the minimum lethal dose has been estimated between 2 and 3 g in infants and between 15 and 20 g in adults [176]. Although there is no Recommended Dietary Allowance (RDA) for boron and its role in human and animal metabolism is not fully understood, it is important to understand boron’s potential in various biologically active scaffolds for strategic drug design and to recognize the proven safety profile of boron and its byproducts at pharmaceutical dosages. Simultaneously, in order to support the incorporation of boron-based compounds in medicine, further studies on boron’s effects on the body are warranted [176].

## 20. Conclusions

Boron has gained momentum in drug discovery over the past few decades due to its unique chemical properties. Boron-containing compounds have shown immense potential, showing their efficacy in clinical phases of drug discovery. Boron’s unique Lewis acidity enables a mechanism of action through the formation of reversible covalent bonds within the active site of target proteins. Further, boron’s low toxicological profile makes it an excellent and sustainable element for drug discovery and development. In addition, boron-containing compounds have demonstrated distinctive biological target engagement, exhibiting effective pharmacodynamic and pharmacological activity and displaying good pharmacokinetic properties. Due to its versatility in biological target engagement, B has become an attractive element for opening new avenues in targeting various disease modalities. Thus, more research is needed to incorporate B into rational drug design to explore its potential in translational research for the benefit of humankind.

## Figures and Tables

**Table 2:** Minimum inhibitory concentrations (MIC) of select Engineered nanomaterials (ENM) in ampicillin-resistant *Escherichia coli*.

Promising ENMs	MIC (µg/mL)	Less Effective ENMs	MIC (µg/mL)
BN	125	SnO_2_	>500
Ag	125	SiO_2_	>500
Ni_2_O_3_	250	Mn_2_O_3_	>500
GO	500	In_2_O_3_	>500
CuO	500	In_2_O_3_	>500
WS_2_	>500	NiO	>500
MoS_2_	>500	CNF	>500
ZnO	>500	CNT	>500

**Table 3:** Comparison of additives and ligands in the borylation of dehydroalanine-containing 2-acetylamino-*N*-benzyl-acrylamide to form Bal. Candidates were tested with Cu(II) source and B_2_(OH)_4_.

Candidate	Type	Yield
2,2′-bipyridine	Bidentate Ligand	6–11%
1,8-*bis*(dimethylamino)naphthalene	Bidentate Ligand	6–11%
Pyridine	Monodentate Pyridine Additive	96–99%
4-picoline	Monodentate Pyridine Additive	96–99%
Imidazole	Monodentate imidazole	66–88%
1-histidine	Monodentate imidazole	66–88%
Guanidine hydrochloride	Protein Denaturant	18%
Urea	Protein Denaturant	0%

**Table 4:** Geometry and boronoalanine (Bal) conversion yields for tested proteins. Conversion yields were measured at various amino acid positions.

Protein	Geometry	Bal Conversion Yield
Histone H3	Small, α-helical	9: 73% 10: >90%
Histone H4	Small, α-helical	16: >95%
pre-SUMO1	Small, globular with α-helices and β-sheets	51: >95%
Npβ	β-helical repeats	61: >95%
mCherry	β barrel	131: 35%
Pantothenate Synthetase (PanC)	Dimer	44: 71% 47: 60%
Modified phosphate-binding protein (PstS)	Globular domains with β-sheets and α-helices	197: 79%
Annexin V	Globular α-helical	316: >95%
Amyloid Precursor Protein (AcrA)	Contains a single helix with a short tail	123: 77%

**Table 5:** Relative accessibility of Bal-protein reactive sites to H_2_O_2_. Note that ROS Concentrations for Npβ-Bal61 and PstS-Bal197 were not specified.

Protein	Scope of Analysis	ROS Concentration	Relative Accessibility
H3-Bal10	1.0–2.8 Å	5 mM	71–108%
H4-Bal16	2.8 Å	20 mM	60%
H3-Bal9	2.8 Å	20 mM	34%
Npβ-Bal61	2.8 Å	–	25
PstS-Bal197	2.8 Å	–	0

## Data Availability

No new data were created or analyzed in this study. Data sharing is not applicable to this article.

## Abbreviations

LscpC: Laspartomycin C; CDA: calcium-dependent antibiotic; MRSA: methicillin-resistant *Staphylococcus aureus*; VISA: vancomycin-intermediate *S. aureus*; VRSA: vancomycin-resistant *S. aureus*; VRE: vancomycin-resistant *enterococci*; Asp: aspartic acid; Ser: serine; Hse: homoserine; PBA: phenylboronic acid; MIC: minimum inhibitory concentrations; MRSE: methicillin-resistant *Staphylococcus epidermidis*; MOA: mechanism of action; MD: molecular dynamics; TLC: thin-layer chromatography; HR-NSE-MS: high-resolution native electrospray ionization mass spectrometry; BA: Boronic acids; pBoF: para-boronophenylalanine; LmrR: Lactococcal multidrug resistance regulatory protein; BOS: boronic-acid (BA)-dependent oxime synthase (BOS); SSM: site-saturation mutagenesis; BMAD: boron-mediated aglycon delivery; PGs: Protecting groups; LanC: Lanatoside C; SPAAC: strain-promoted azide–alkyne cycloaddition; DBCO: dibenzocyclooctyne; HCT116: Human Colorectal Carcinoma Cell line; AZM: azithromycin; TEL: telithromycin; NTM: nontuberculous mycobacteria; cryo-EM: cryogenic electron microscopy; NPET: nascent peptide exit tunnel; LeuRS: leucyl-tRNA synthetase; ITC: titration calorimetry; VL: Visceral leishmaniasis; Sbv: pentavalent antimonial; SSG: sodium stibogluconate; MA: meglumine antimoniate; PM: paromomycin; MF: miltefosine; AmpB: amphotericin B; HAT: human African trypanosomiasis; MoA: mode of action; CPSF3: Cleavage and Polyadenylation Specificity Factor 3; AD: Alzheimer’s disease; Aβ: β-amyloid; MIDA: N-methyliminodiacetate; PDE: phosphodiesterase; BA: Boronic-acid; ABH: (S)-2-amino-6-boronohexanoic acid; WHO: World Health Organization; NHP: non-human primates; SARs: structure-activity relationships; PET: positron-emission tomography; BPA: boronophenylalanine; BoPs: boronated phospholipids; MD: Molecular dynamics; SRB: Sulforhodamine B; PARP1: Poly (ADP-ribose) polymerase-1; ENMs: engineered nanomaterials; AMR: antimicrobial-resistant; TEM: transmission electron microscopy; AFM: atomic force microscopy; Ag NPs: silver nanoparticles; BN: Boron Nitride; BMDCs: bone marrow-derived dendritic cells; H&E: Hematoxylin and Eosin staining; SRM: super-resolution microscopy imaging; BLSP: biotin labelling of the surface proteome; CNF: cellulose nanofibers; C: carbon; Si: silicon; B: Boron; LABP: Lewis acid-base pairing; O: oxygen; N: nitrogen; H: hydrogen (H); H_2_O: water; MiTyRS: Methanococcus jannaschii tyrosyl-tRNA synthetase; Dha: dehydroalanine; B2(OH)4: tetrahydroxyboron; Bal: boronoalanine; Nap: Sodium Phosphate buffer; PanC: Pantothenate Synthetase; ROS: reactive oxygen species; TON: onset temperature; TM: melting temperature; SUMO1: Small ubiquitin-like modifier; PRE: Paramagnetic relaxation enhancement; DICE: Dative-contact induced chemical exchange; RDA: Recommended Dietary Allowance; WHO: World Health Organization.
